# Toward understanding the catalytic mechanism for two classes of bacterial phospholipid *N*‐methyltransferases

**DOI:** 10.1111/febs.70571

**Published:** 2026-05-02

**Authors:** Irina Shevyreva, Lena Sophie Fritsch, Leander Mika Koch, Anton Effing, Meriyem Aktas, Franz Narberhaus

**Affiliations:** ^1^ Microbial Biology, Faculty of Biology and Biotechnology Ruhr University Bochum Germany

**Keywords:** bacterial membrane, catalytic mechanism, phosphatidylcholine, phospholipid, phospholipid *N*‐methyltransferase

## Abstract

Phosphatidylcholine (PC), the predominant phospholipid in eukaryotic membranes, also plays a crucial role in certain bacterial species, often mediating interactions with eukaryotic hosts. In bacteria, a major pathway for PC biosynthesis involves the three‐step methylation of phosphatidylethanolamine, catalyzed by phospholipid *N*‐methyltransferases (Pmts). While the binding site for the methyl donor *S*‐adenosyl‐*l*‐methionine is well characterized in Pmt enzymes, the detailed mechanism of methyl group transfer remains poorly understood. In this study, we combined computational and biochemical approaches to identify the amino acid residues critical for the catalytic activity of two distinct Pmt classes: the *Rhodobacter* (*R*)‐type enzyme from *Rubellimicrobium thermophilum* (*Rt*PmtA) and the *Sinorhizobium* (*S*)‐type enzyme from *Agrobacterium tumefaciens* (*At*PmtA). Despite low sequence identity, both enzyme types share similar reaction mechanisms, with tyrosine residues playing key roles in methyl group transfer. In *Rt*PmtA, two highly conserved tyrosines located within the substrate‐binding pocket on the N‐terminal αA‐helix are critical for enzymatic function. In contrast, *At*PmtA depends on a single tyrosine buried in the protein core for catalysis. These findings reveal distinct active site architectures and suggest that *R*‐type and *S*‐type enzymes have evolved class‐specific structural strategies for tyrosine activation. This divergence highlights the evolutionary flexibility of Pmt enzymes, despite their shared catalytic function.

## Graphical abstract

Phospholipid *N*‐methyltransferases (Pmts) synthesize phosphatidylcholine in many bacteria. In this study, we compared the catalytic mechanisms of two bacterial Pmt classes: the *Rhodobacter* (*R*‐) and the *Sinorhizobium* (*S*‐) type. Representative enzymes for each class were derived from *Rubellimicrobium thermophilum* and *Agrobacterium tumefaciens*, respectively. Our results indicate that tyrosine (Y) residues play a critical role in the catalytic mechanisms of both enzymes, although their modes of activation differ.

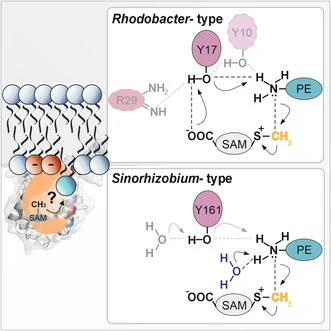

**Abbreviations**
CD spectroscopy: circular dichroism spectroscopy
DMPE: dimethyl‐phosphatidylethanolamine
EV: empty vector
MMPE: monomethyl‐phosphatidylethanolamine
PC: phosphatidylcholine
PE: phosphatidylethanolamine
PG: phosphatidylglycerol
Pmt: phospholipid *N‐methyltransferase*
*R*‐type: *Rhodobacter*‐type
SAH: *S*‐adenosyl‐*l*‐homocysteine
SAM: *S*‐adenosyl‐*l*‐methionine
*S*‐type: *Sinorhizobium*‐type
TLC: thin‐layer chromatography
WT: wild‐type

## Introduction

Phosphatidylcholine (PC), the predominant phospholipid in eukaryotic membranes, is also found in approximately 15% of bacterial species [1, 2]. While some bacteria produce only trace amounts, in others, PC is a major membrane component. For instance, PC accounts for just a few percent of total membrane lipids in *Xanthomonas campestris*, whereas it can comprise up to 75% in *Acetobacter aceti* [3, 4]. Due to its cylindrical shape, PC contributes to the formation of stable lipid bilayers, enhancing membrane integrity and resilience under environmental stress, such as extreme temperatures, the presence of the anionic detergent sodium dodecyl sulfate (SDS), or acetic acid [3, 5].

Beyond its structural role, PC is critical for mediating interactions between bacteria and their eukaryotic hosts, in both pathogenic and symbiotic contexts. For instance, the plant–pathogen *Agrobacterium tumefaciens* requires PC to induce crown gall tumors on plants [5, 6]. Similarly, a PC‐deficient strain of the human pathogen *Brucella abortus* displays reduced virulence in mice [7]. PC is equally important in symbiotic interactions. In *Bradyrhizobium diazoefficiens* (formerly *B. japonicum*), reduced PC amounts impair root nodule development and nitrogen fixation of soybean hosts [8]. Likewise, *Rhizobium leguminosarum* fails to establish an effective symbiosis with clover in the absence of PmtS2, a key enzyme in PC biosynthesis [9].

One of the primary pathways for PC biosynthesis in bacteria is the methylation pathway (Fig. 1). This route involves one or more phospholipid *N*‐methyltransferase (Pmt) enzyme(s) that work together to synthesize PC (Fig. 2). These cytosolic enzymes transiently associate with the membrane to carry out their function. Pmts catalyze a series of three sequential methylation reactions on the substrate phosphatidylethanolamine (PE), generating monomethyl‐PE (MMPE), dimethyl‐PE (DMPE), and ultimately PC (Fig. 1). Each methylation step uses *S*‐adenosyl‐*l*‐methionine (SAM) as the methyl donor, yielding *S*‐adenosyl‐*l*‐homocysteine (SAH) [10, 11, 12].

**Fig. 1:**
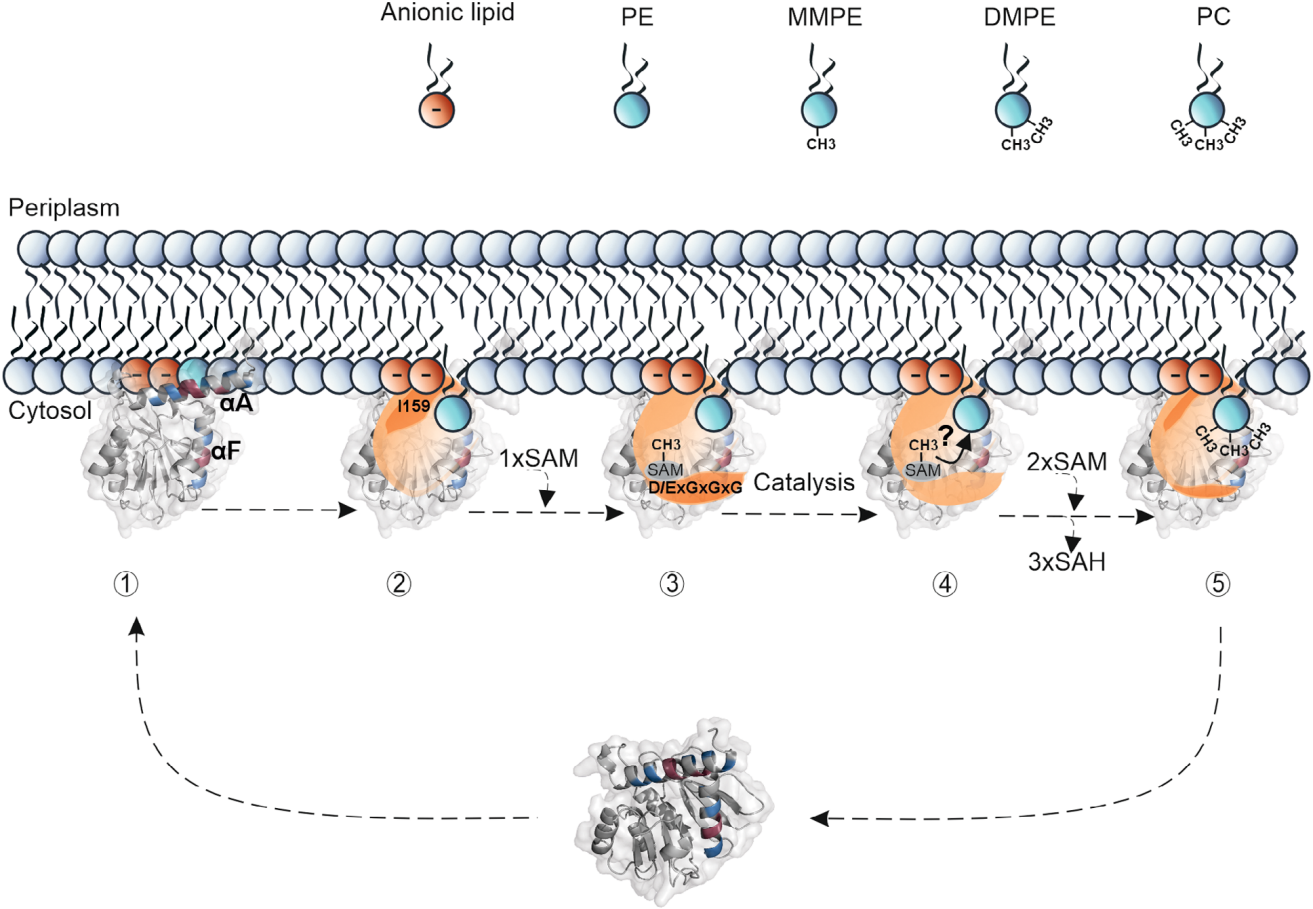
Proposed catalytic cycle of bacterial phospholipid *N*‐methyltransferases (Pmts), exemplified by *A. tumefaciens* PmtA (AlphaFold 3 model). (1) Membrane association, primarily via an N‐terminal amphipathic αA and C‐terminal αF helices, occurs at membrane regions enriched in negatively charged lipids. (2) Lipid‐substrate (PE) binding is facilitated by its partial extraction from the membrane bilayer. Isoleucine (I159) was shown to be responsible for the substrate specificity of *At*PmtA. (3) Following the lipid‐substrate‐binding, SAM binds at the (D/E)xGxGxG motif. (4) Methyl group transfer, the primary focus of this study, occurs once both substrate and cofactor are correctly positioned. (5) Formation of the end product PC is followed by enzyme dissociation from the membrane. The enzyme undergoes a series of sequential conformational changes (highlighted in orange) throughout steps 1–5. DMPE, dimethyl‐PE; MMPE, monomethyl‐PE; PC, phosphatidylcholine; PE, phosphatidylethanolamine; SAH, *S*‐adenosylhomocysteine; SAM, *S*‐adenosylmethionine. Adapted and modified from publications by Danne *et al*. [11], Salsabila and Kim [13], Watanabe *et al*. [14].

**Fig. 2:**
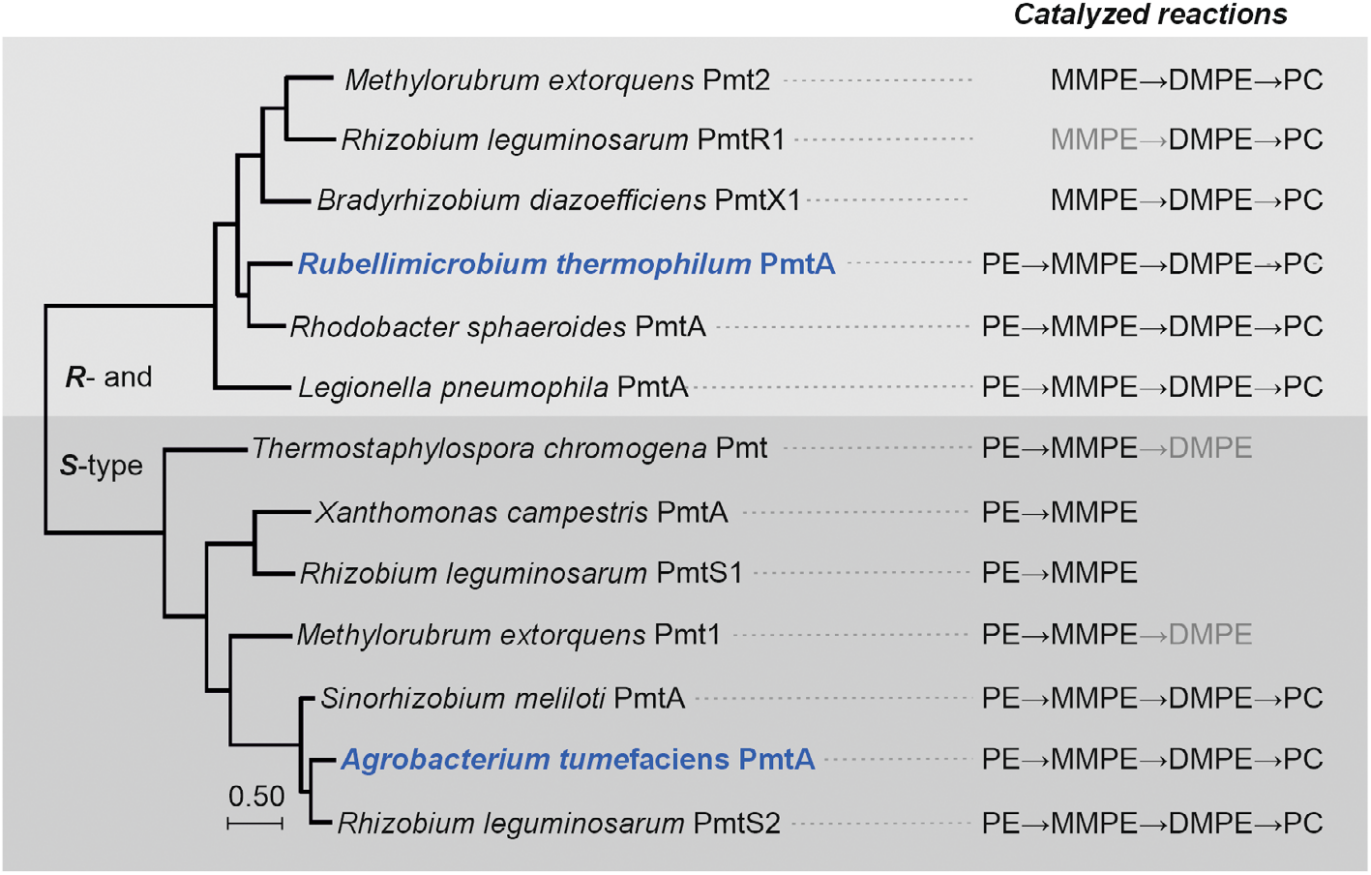
Phylogenetic tree of bacterial phospholipid *N*‐methyltransferases (Pmts) with their classification into *Rhodobacter* (*R*) and *Sinorhizobium* (*S*) types. *R. thermophilum* PmtA and *A. tumefaciens* PmtA were selected as representative proteins for each type and are highlighted in blue. Catalyzed reactions are indicated to the right of the respective enzyme. Sequence analysis was performed using the megax software [15, 16]. The distance values show the number of substitutions as a proportion of the alignment's length. NCBI identification numbers: *R*‐type: *Methylorubrum extorquens* Pmt2 (ACS42118; [17, 18], *Rhizobium leguminosarum* PmtR1 (DLJ82_4359; [9]), *Bradyrhizobium diazoefficiens* PmtX1 (BAC52259; [19]), *Rubellimicrobium thermophilum* PmtA (Ruthe_01610; [20]), *Rhodobacter sphaeroides* PmtA (Q05197; [21]), *Legionella pneumophila* PmtA (WP_010947869; [22]). *S*‐type: *Thermostaphylospora chromogena* Pmt (SAMN04489764_3900; [20]), *Xanthomonas campestris* PmtA (CAP49368; [4]), *R. leguminosarum* PmtS1 (DLJ82_3449; [9]), *M. extorquens* Pmt1 (ABY31351; [17]), *Sinorhizobium meliloti* PmtA (Q92SN7; [23]), *A. tumefaciens* PmtA (AAK86115; [10]), *R. leguminosarum* PmtS2 (DLJ82_3306; [9]).

Bacterial Pmts are classified into two major families—*Rhodobacter* (*R*)‐type and *Sinorhizobium* (*S*)‐type—based on amino acid sequence homology (Fig. 2). Although these enzymes perform the same biological function and share a conserved overall topology, including the characteristic Rossmann fold with the highly conserved SAM‐binding motif [(D/E)xGxGxG], *R*‐ and *S*‐type Pmts display less than 20% sequence identity [13, 24, 25]. Recent crystallographic analyses of *Rt*PmtA from *Rubellimicrobium thermophilum* (*R*‐type) and *At*PmtA from *A. tumefaciens* (*S*‐type) have confirmed that, despite their low sequence similarity, both enzyme types share key structural features [13, 14].

Previous studies suggest that three initial steps likely associated with conformational changes are required before Pmts can effectively transfer a methyl group to PE (Fig. 1). Although bacterial Pmts are cytosolic, they transiently associate with the cytoplasmic membrane via amphipathic helices, engaging in electrostatic interactions with negatively charged phospholipids, such as phosphatidylglycerol (PG) or cardiolipin (CL) [11, 26]. For example, the *S*‐type *At*PmtA uses both the amphipathic αA and αF helices for membrane binding, with the N‐terminal αA‐helix showing stronger affinity (Fig. 1) [11, 26]. Similarly, the *R*‐type PmtX1 from *B. diazoefficiens* interacts with the membrane via its N‐terminal αA‐helix, while *Rt*PmtA employs its C‐terminal α8 and α9 helices for membrane attachment [13, 26].

Following membrane association, Pmts bind their lipid substrate, which in turn allows access to the SAM‐binding pocket (Fig. 1). The binding events conform to an ordered Bi‐Bi reaction model, in which substrate‐binding precedes cofactor association [10, 27]. In the case of *At*PmtA, SAM binding occurs only in the presence of lipid substrates [10], and is significantly enhanced when negatively charged lipids—such as PG or CL—are also present [11]. Moreover, substrate specificity in *At*PmtA is defined by a branched isoleucine residue at position 159, which enables the enzyme to accept and convert the smallest lipid substrate, PE, as well as the bulkier methylated lipids MMPE and DMPE (Fig. 1, step 2) [14]. These findings suggest that anionic lipids promote both substrate and SAM binding, likely by inducing conformational changes in the enzyme. Structural analysis of *Rt*PmtA supports this model, revealing a subtle helix transition upon lipid‐substrate‐binding and stimulation of enzymatic activity in the presence of PG, as previously demonstrated *in vitro* [13, 20]. Despite these advances in our biochemical and structural understanding of Pmts, the precise catalytic mechanism of methyl group transfer—particularly the identity and role of key amino acid residues involved—remains not fully understood (Fig. 1, step 4).

In general, SAM‐dependent methyltransferases catalyze the transfer of a methyl group from the donor molecule SAM to a carbon, oxygen, sulfur, or nitrogen atom on the acceptor molecule. This process involves a series of conformational changes, including reorientation of surrounding amino acids to properly align the substrate and SAM. Once the optimal geometry is achieved, the reaction proceeds via an S_N_2‐type nucleophilic attack on the methyl group connected to the positively charged sulfur atom of SAM. This leads to cleavage of the C—S bond and successful transfer of the methyl group to the acceptor [28].

In this study, we present a comparative in‐depth analysis of two representative Pmts: the *S*‐type *At*PmtA from *A. tumefaciens* and the *R*‐type *Rt*PmtA from *R. thermophilum*. Although both enzymes catalyze the stepwise methylation of PE to MMPE, DMPE and PC (Fig. 2), they share only ~ 30% sequence similarity, as determined using the Ident and Sim webserver [29]. By integrating biochemical assays with computational analyses (sequence alignments, structural modeling, pocket predictions, and substrate docking), we identified several conserved tyrosine residues likely involved in catalysis. Notably, the number and spatial arrangement of these catalytic residues differ between the two enzyme types. *R‐*type *Rt*PmtA contains two highly conserved tyrosines located within the substrate‐binding pocket on the N‐terminal αA‐helix, whereas *S*‐type *At*PmtA features a single tyrosine buried within the protein core. Based on these findings, we propose two distinct yet functionally convergent catalytic mechanisms for *R*‐ and *S*‐type Pmts, underscoring their structural divergence and adaptive evolutionary strategies. During the course of our investigation, two studies were published that reported high‐resolution crystal structures of these enzymes [13, 14]. These structural snapshots of the catalytic cycle corroborate and extend our findings, providing additional support for the proposed mechanism of the enzyme's function (Fig. 1).

## Results

### Identification of amino acid candidates important for catalytic activity of *R*‐ and *S*‐type Pmts

To better understand the catalytic mechanism of Pmt enzymes, we conducted a comprehensive literature review on established methylation strategies [28, 30, 31]. Three principle catalytic mechanisms have been described for SAM‐dependent methyltransferases: (a) the proximity and desolvation mechanism [32], (b) the acid/base‐mediated mechanism [33] and (c) the metal‐dependent mechanism [34, 35]. In the proximity and desolvation mechanism, the active site does not contribute catalytically to methyl group transfer through specific residues. Instead, it provides a structurally suitable environment that aligns the lipid substrate and SAM in close proximity while excluding water molecules, thereby facilitating efficient methyl group transfer. The acid/base‐mediated mechanism, by contrast, involves charged amino acids strategically positioned to act as general acids or bases. These residues can deprotonate the substrate, increasing its nucleophilicity and thereby promoting a nucleophilic attack on the methyl group of SAM. Tyrosine residues have been implicated in both this and the proximity/desolvation model. The third, metal‐dependent mechanism is primarily observed in phenolic *O*‐methyltransferases in plants. In this case, metal ions act as general bases by modulating the pK_a_ of phenolic groups, thereby facilitating methylation. Since neither *Rt*PmtA nor *At*PmtA requires additional metal ions for catalytic activity, the metal‐dependent mechanism was excluded from further consideration.

To identify conserved amino acids potentially involved in catalysis, we performed a multiple sequence alignment of known Pmts using the clustalW algorithm implemented in the megax software [15, 36]. A phylogenetic tree was generated using the maximum likelihood method (Fig. 2). Based on their demonstrated catalytic competence in previous studies—specifically, their ability to catalyze all three methylation reactions [10, 20]—we selected the *R*‐type *Rt*PmtA from *R. thermophilum* and the *S*‐type *At*PmtA from *A. tumefaciens* for further structural and functional analysis.

Tyrosine residues are known to be involved in both the proximity/desolvation and acid/base‐mediated mechanisms, along with positively and negatively charged amino acids present mainly in the latter one [32, 33, 37]. Since highly conserved residues often play critical roles in protein function and catalysis [38, 39], we prioritized such residues as likely candidates essential for enzymatic activity in both Pmt types (Figs S1 [13, 40, 41] and S2 [11, 14, 40, 41]). To refine our analysis, we conducted a groupwise sequence alignment using clustal omega [40], and mapped conserved residues onto available 3D structures or AlphaFold 3‐predicted models of related enzymes. To further narrow down potential catalytic residues, we applied the P2Rank ligand‐binding pocket prediction tool [41, 42, 43], which enabled the exclusion of conserved residues located outside the predicted active site. Only highly conserved residues localized within the predicted substrate‐binding pocket were selected for further functional analysis.

In addition, we performed a comparative analysis of experimentally determined structures and AlphaFold 3 models (Figs S3A [13, 40, 41] and S4A [14, 40, 41]), including a direct comparison of the predicted substrate‐binding pockets (Figs S3B [13, 40, 41] and S4B [14, 40, 41]). Overall, the predicted models and validated structures for both, *Rt*PmtA and *At*PmtA, were highly similar, with only minor differences observed in the predicted substrate‐binding pocket.

Based on the previously mentioned selection criteria, the candidate residues identified in *Rt*PmtA were Y10, Y17, D18, D73, D99, H147, and E160, while those in *At*PmtA were Y161, E84, and D106 (Figs 3A and 6A). To evaluate the functional importance of these residues in methyl group transfer, we conducted site‐directed mutagenesis. Conserved tyrosine residues located within the predicted substrate‐binding pockets were substituted with alanine, phenylalanine or serine to assess the influence of side‐chain size and the presence of a hydroxyl group. Negatively charged residues (aspartate and glutamate) were replaced with polar, uncharged analogs, while the positively charged histidine residues were substituted with alanine and phenylalanine to probe their potential catalytic roles.

**Fig. 3:**
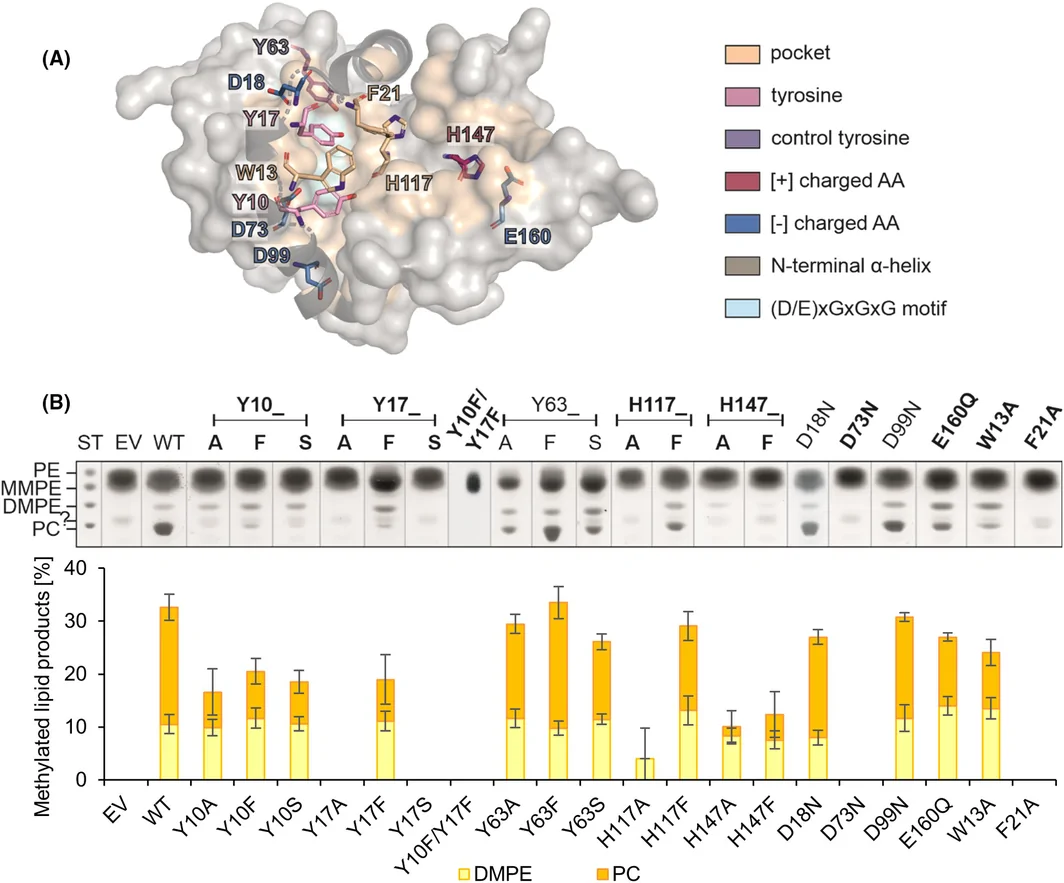
Putative catalytic residues in *R*‐type *R. thermophilum* PmtA and *in vivo* activity of mutated variants. (A) Crystal structure of *Rt*PmtA solved by Salsabila and Kim [13] with candidate amino acids (AA) potentially involved in catalysis. The predicted substrate‐binding pocket, identified using p2rank [41, 42], is shown in orange. Two highly conserved tyrosines (pink) on the N‐terminal α‐helix (gray), one positively [+] charged (red) and three negatively [−] charged amino acids (blue) identified via sequence analysis are also located in the predicted pocket. A control tyrosine (purple) was included due to its high conservation and proximity to the predicted active site. Additional residues W13, F21, and H117 (highlighted in orange) were previously proposed to contribute to catalysis Salsabila and Kim [13]. The *S*‐adenosyl‐*l*‐methionine (SAM)‐binding motif (D/E)xGxGxG is shown in light blue. (B) *In vivo* activity of wild‐type (WT) *Rt*PmtA and protein variants in *E. coli* NiCo21 after 4 h of incubation at 30 °C. Lipids were extracted and analyzed via TLC. Copper sulfate staining was used to visualize and quantify lipid levels based on band intensity. Images are shown in grayscale. Data represent mean values from two or more independent experiments; error bars indicate standard deviation. ‘?’, an unknown lipid; DMPE, dimethyl‐PE; EV, empty vector; MMPE, monomethyl‐PE; PC, phosphatidylcholine; PE, phosphatidylethanolamine; ST, lipid standard; WT, wild type protein. The variants with abolished or reduced activity are shown in bold above TLC plate.

In addition, several *Rt*PmtA variants, namely H117A/F, W13A, and F21A, were generated based on prior structural and biochemical evidence indicating their potential involvement in catalysis [13]. To further validate our selection criteria, we included residues Y63 in *Rt*PmtA and Y85 in *At*PmtA in the mutational analysis. While Y63 is highly conserved among *R*‐type Pmts, it is not expected to reside within the substrate‐binding pocket. In contrast, Y85 is located within the putative active site of *At*PmtA yet lacks strong conservation across *S*‐type Pmts (Figs S1 [13, 40, 41] and S2 [11, 14, 40, 41]).

### *In vivo* activity of *Rt*PmtA variants

For heterologous production and *in vivo* activity characterization of the *R*‐type *Rt*PmtA and *S*‐type *At*PmtA variants, *E. coli* was used as the host organism. Due to its native lipid composition comprising primarily PE, PG, and CL, *E. coli* provides a suitable environment for the functional analysis of PC‐synthesizing enzymes [2, 17]. Successful expression of all Pmt variants was confirmed by SDS/PAGE and western blot, which showed a protein band at the expected molecular mass of ~ 25 kDa, absent in the empty vector (EV) control (Fig. S5). To assess the catalytic activity of the *Rt*PmtA variants, the levels of methylated lipid products were quantified by analyzing band intensities using the imagej software (Table S1 [44]).

As shown in Fig. 3B, the lipid profile of *E. coli* producing recombinant wild‐type (WT) *Rt*PmtA revealed substantial levels of DMPE (11%) and PC (22%), in contrast to the EV control. Consistent with previous findings [9], the EV control displayed a prominent band composed of PE, PG, and CL, which co‐migrate in the employed solvent system and were present in all samples. A faint band of unknown identity observed between DMPE and PC was also noted across samples.

For the quantitative product analysis of protein variants, DMPE and PC levels were set to 0% when the lipid profile closely resembled that of the EV control, indicating a lack of enzymatic activity. Variants harboring mutations in residues presumed to be catalytically irrelevant were expected to retain WT‐like activity. Consistent with this expectation, the Y63A/F/S, D18N, and D99N variants exhibited lipid profiles comparable to that of WT *Rt*PmtA. Similarly, H117F maintained near‐native activity, whereas H117A showed markedly reduced function (Fig. 3B and Table S1 [44]).

Most of the tested variants (Y10A/F/S, Y17F, H147A/F, E160Q, and W13A) displayed reduced activity, while variants Y17A/S, D73N, and F21A, as well as the Y10F/Y17F protein were completely inactive (Fig. 3B and Table S1 [44]). Based on these results, we selected only those variants exhibiting significantly diminished or abolished activity that were selected for further *in vitro* biochemical characterization.

### *In vitro* activity of *Rt*PmtA variants

His‐tagged *Rt*PmtA variants of interest were purified (Fig. S6A) and subjected to *in vitro* activity assays. The key advantage of the *in vitro* setup was the ability to test different substrate combinations, both in the presence and absence of the anionic lipid PG (Fig. S7 and Table S2 [44]). Since anionic lipids stimulate the activity of both *Rt*PmtA and *At*PmtA [10, 20], only samples supplemented with PG were used for quantitative analysis, with product formation evaluated by band intensity.

Figure 4A demonstrates that in the presence of PE/PG, only WT *Rt*PmtA and the variants Y10A, H117F, E160Q, and W13A retained residual activity, as indicated by detectable DMPE and, in some cases, PC production. All other variants failed to convert PE under these conditions, indicating a complete loss of activity (Fig. S7 and Table S2 [44]).

**Fig. 4:**
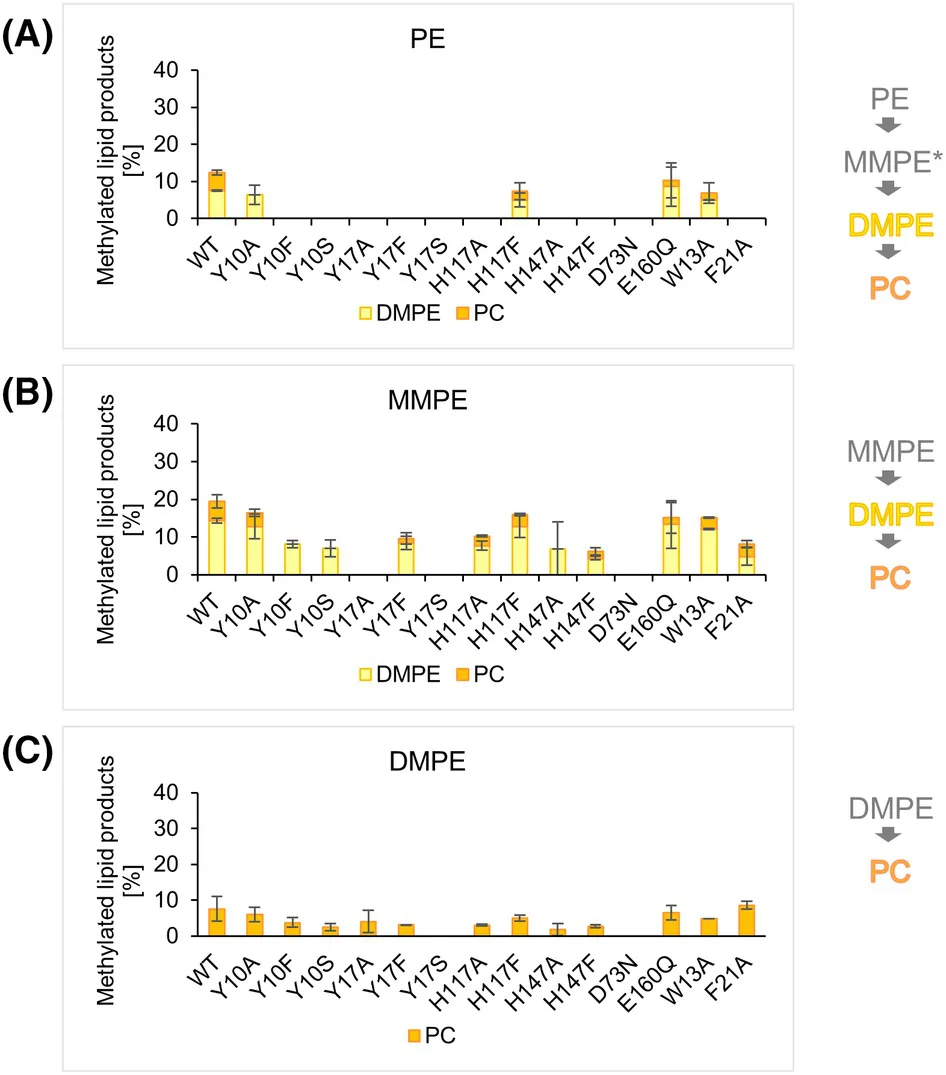
Evaluation of *in vitro* activity of *Rt*PmtA with different lipid substrates. Phosphatidylethanolamine (PE) (A), monomethyl‐PE (MMPE) (B), and dimethyl‐PE (DMPE) (C) were used as lipid substrates, and phosphatidylglycerol (PG) as anionic lipid required for full activity. The relative levels of the methylated PE products DMPE and PC after 4 h of reaction time were quantified by thin‐layer chromatography (TLC) analysis using imagej (Fig. S7 and Table S2 [44]). The total lipid amount of each sample was set to 100%. MMPE* was left out of quantification because of its retention behavior. The data shown represent the mean values of two or more independent measurements. Error bars indicate standard deviation.

In contrast, the ability to methylate MMPE to DMPE and PC was retained in most protein variants (Fig. 4B). While WT *Rt*PmtA converted MMPE to 14% DMPE and 5% PC, similar levels were observed for Y10A, H117F, E160Q, and W13A. Variants Y17F, H117A, H147F, and F21A showed residual activity. In contrast, Y10F, Y10S, and H147A produced only DMPE, but no PC. Notably, Y17A, Y17S, and D73N were completely inactive, showing no MMPE methylation under the assay conditions (Fig. S7 and Table S2 [44]).

When DMPE was used as substrate, all *Rt*PmtA variants, except for Y17S and D73N, could produce PC (Fig. 4C). Among them, E160Q and F21A produced PC levels comparable to the WT, whereas Y10A, H117F, and W13A variants showed reduced PC production. Moreover, Y10F/S, Y17A/F, H117A, and H147A/F generated only trace amounts of PC. As with the other substrates, Y17S and D73N variants were completely inactive (Fig. S7 and Table S2 [44]). Overall, the results indicate that most variants displayed greater catalytic activity with MMPE and DMPE than with PE.

### Secondary structure analysis of *Rt*PmtA variants

To determine whether the reduced enzymatic activity observed in some *Rt*PmtA variants was due to specific amino acid substitutions in the active site, rather than general protein misfolding, we employed CD spectroscopy to assess their secondary structure integrity. In addition, to exclude the possibility that the loss of activity resulted from impaired membrane association, we evaluated the membrane‐binding capacity of the proteins. Specifically, CD spectra were recorded in the presence and absence of PG liposomes. Binding of the protein to liposomes is expected to induce a detectable shift in the CD spectrum, reflecting conformational changes upon membrane interaction [45].

CD spectral analysis of WT *Rt*PmtA and nearly all tested variants confirmed proper protein folding (Figs 5A and S8A). The spectra consistently displayed negative peaks at wavelengths near 208 and 222 nm, along with a negative peak around 218 nm and a positive peak near 195 nm, characteristic of proteins containing a mixture of α‐helical and β‐strand elements [46]. This spectral profile is consistent with previously reported data for *At*PmtA [24]. Almost all analyzed variants showed CD spectra comparable to the WT protein, indicating preserved secondary structure. Only the D73N variant exhibited notable deviations, with less pronounced negative peaks, despite being analyzed at the same protein concentration (Fig. S8A). To quantify secondary structure elements, we used the BeStSel™ webserver [47, 48, 49] based on the obtained CD spectra (Table S3, [47, 48, 49]). The analysis revealed that the D73N variant had a marked reduction in α‐helical content compared with the WT protein, suggesting that the mutation may compromise the structural integrity and contribute to its complete loss of enzymatic activity both *in vivo* and *in vitro* (Figs 3 and 4).

**Fig. 5:**
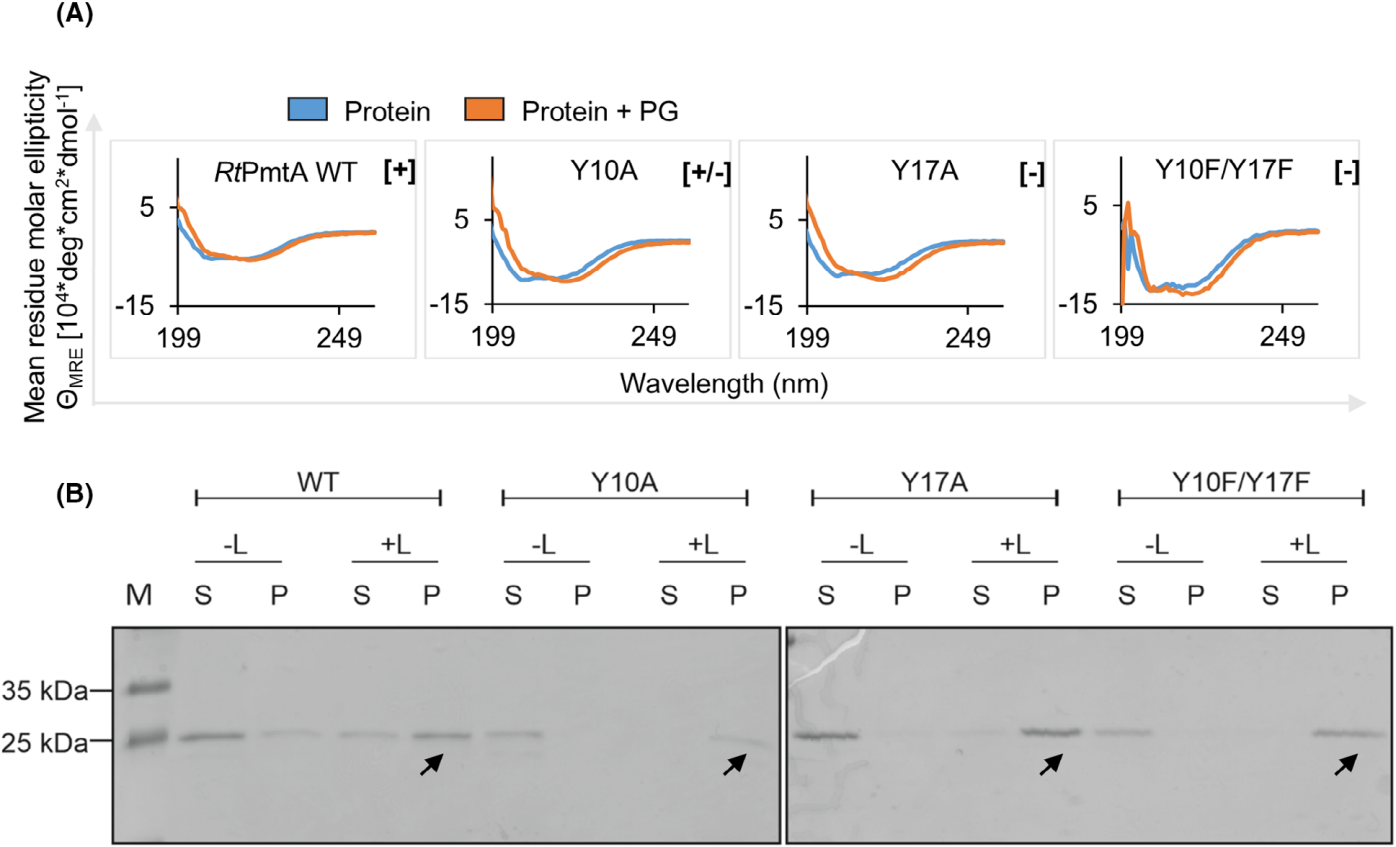
Secondary structure analysis of *Rt*PmtA protein variants in the presence and absence of phosphatidylglycerol (PG) liposomes via circular dichroism (CD) spectroscopy (A) as well as co‐sedimentation assay with phosphatidylethanolamine (PE), PG, and CL‐containing liposomes (B). Filled arrows indicate protein behavior in the presence of liposomes. The CD data shown represent the averaged spectra of two or more independent measurements. *In vivo* activity of the protein variants is indicated as follows: [+]: WT‐like activity, [+/−]: reduced activity, [−]: no activity.

### Lipid binding of *Rt*PmtA variants

All tested Pmt variants, except for D73N, exhibited spectral shifts in the presence of PG liposomes (Figs 5A and S8A). These shifts, though varying in intensity and spectral pattern, indicate that most of the variants retained the ability to associate with anionic lipids, which is a prerequisite for the catalytic cycle (Fig. 1). The variability in the spectral responses suggests subtle conformational differences in membrane interaction among the variants.

To corroborate lipid‐binding activity, liposome co‐sedimentation assays were performed using selected *Rt*PmtA proteins. Specifically, tyrosine variants exhibiting reduced or abolished enzymatic activity were examined for their ability to bind PE : PG : CL‐containing liposomes. Wild‐type *Rt*PmtA served as positive control. In the absence of liposome, this protein was predominantly detected in the supernatant fraction, whereas in the presence of liposomes, it shifted to the pellet fraction (indicated by an arrow in Fig. 5B). All tested variants displayed comparable or even stronger membrane association, with little to no protein remaining in the supernatant when liposomes were present. These results indicate that the *Rt*PmtA variants retained membrane‐binding capacity, demonstrating that the observed reduction or loss of enzymatic activity was not due to impaired membrane association.

### *In vivo* activity of *At*PmtA variants

The *in vivo* activity of the *At*PmtA WT protein and its variants was assessed using the same approach as for *Rt*PmtA (Figs 6 and S5B). Variants at the control tyrosine (Y85) and the D106N mutant exhibited WT‐like activity, producing approximately 6% DMPE and 12% PC. The Y161F retained residual methyltransferase activity, generating around 4% DMPE and 7% PC. In contrast, the Y161A, Y161S, and E84Q variants showed no detectable activity, similar to the ∆N30 control, which lacks the N‐terminal membrane‐binding helix (Fig. 6B and Table S4, [44]). This ∆N30 protein was previously shown to be deficient in membrane association [11].

**Fig. 6:**
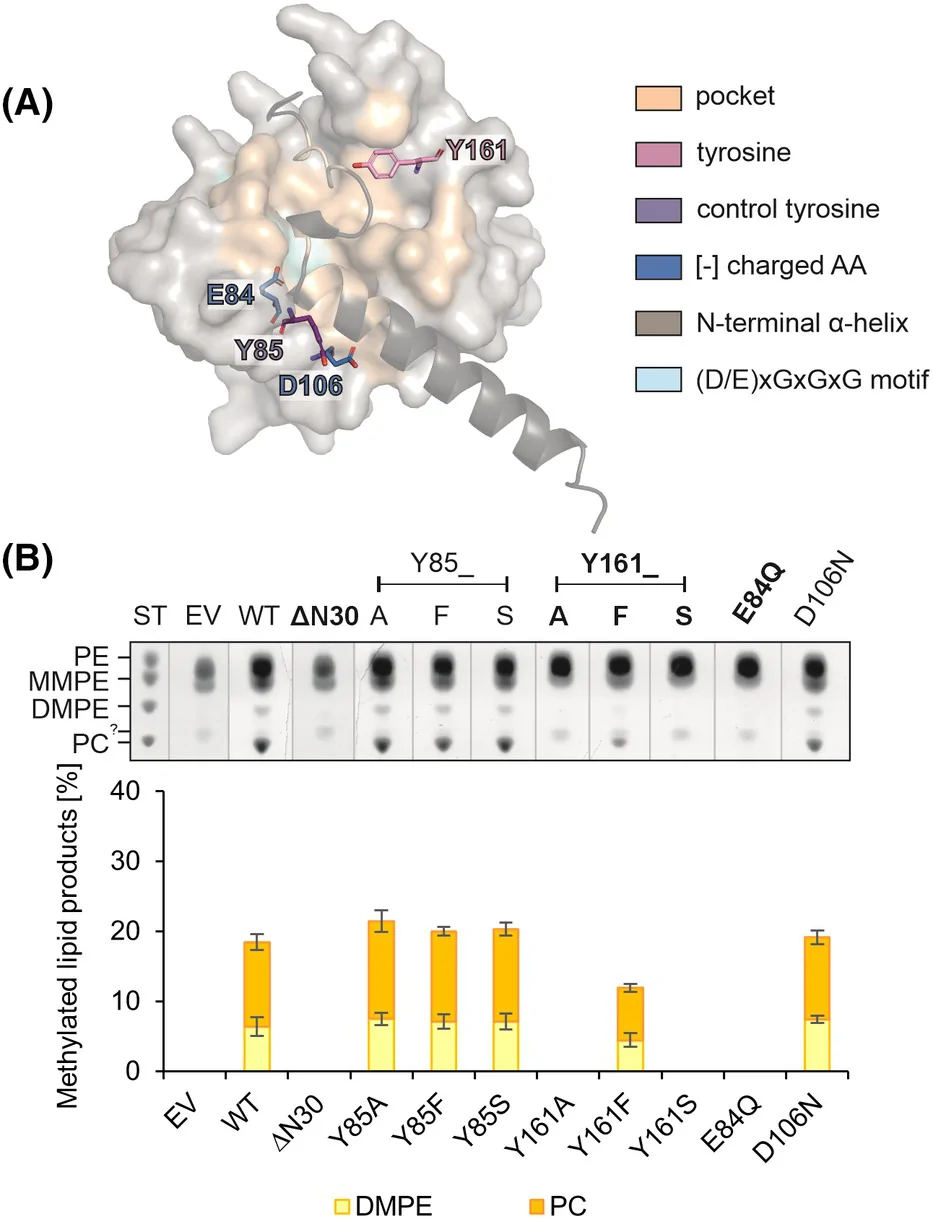
Amino acid candidates potentially responsible for catalytic activity of *A. tumefaciens* PmtA (*At*PmtA). (A) AlphaFold 3 model of *At*PmtA with highlighted amino acid residues possibly essential for catalytic activity of the protein. The pocket predicted by p2rank [41, 42] analysis (orange) contains one highly conserved tyrosine (pink) in the protein core and two negatively [−] charged amino acids (blue). In contrast, additionally selected control tyrosines (purple) were either highly conserved among phospholipid *N*‐methyltransferases (Pmts) or localized in the expected pocket. The N‐terminal αA‐helix is displayed in gray, while the *S*‐adenosyl‐*l*‐methionine (SAM)‐binding motif (D/E)xGxGxG is depicted in light blue. (B) *In vivo* activity assessment of wild‐type *At*PmtA and protein variants after 4 h based on thin‐layer chromatography (TLC). Copper sulfate staining was used to visualize extracted lipids, which were then quantified using the imagej software. Images are presented in grayscale. Lipids that were not formed upon methylation were categorized as other lipids. As a negative control, a truncated form of *At*PmtA ∆N30 with the first 30 amino acids deleted from the N‐terminal αA‐helix was employed. The data shown represent the mean values of two or more independent measurements. MMPE* was left out of quantification because of its retention behavior. Error bars display standard deviation. The variants with abolished or reduced activity are highlighted in bold.

### *In vitro* activity of *At*PmtA variants

The *in vitro* activity of the purified *At*PmtA variants (Fig. S6B) was initially tested using PE as substrate in the presence of PG. Under these conditions, only the WT protein was able to catalyze the formation of DMPE and PC, whereas none of the tested variants produced detectable amounts of these methylated products (Figs 7A, S9 and Table S5, [44]). A similar product profile was observed when MMPE was used as substrate. However, in the presence of DMPE, not only the WT protein produced PC (13% PC), but also three additional variants, Y161A, Y161F, and Y161S, exhibited low levels of DMPE to PC conversion, with yields ranging from 3 to 6% (Figs 7B,C, and S9). The ∆N30 and E84Q variants remained inactive under all tested conditions.

**Fig. 7:**
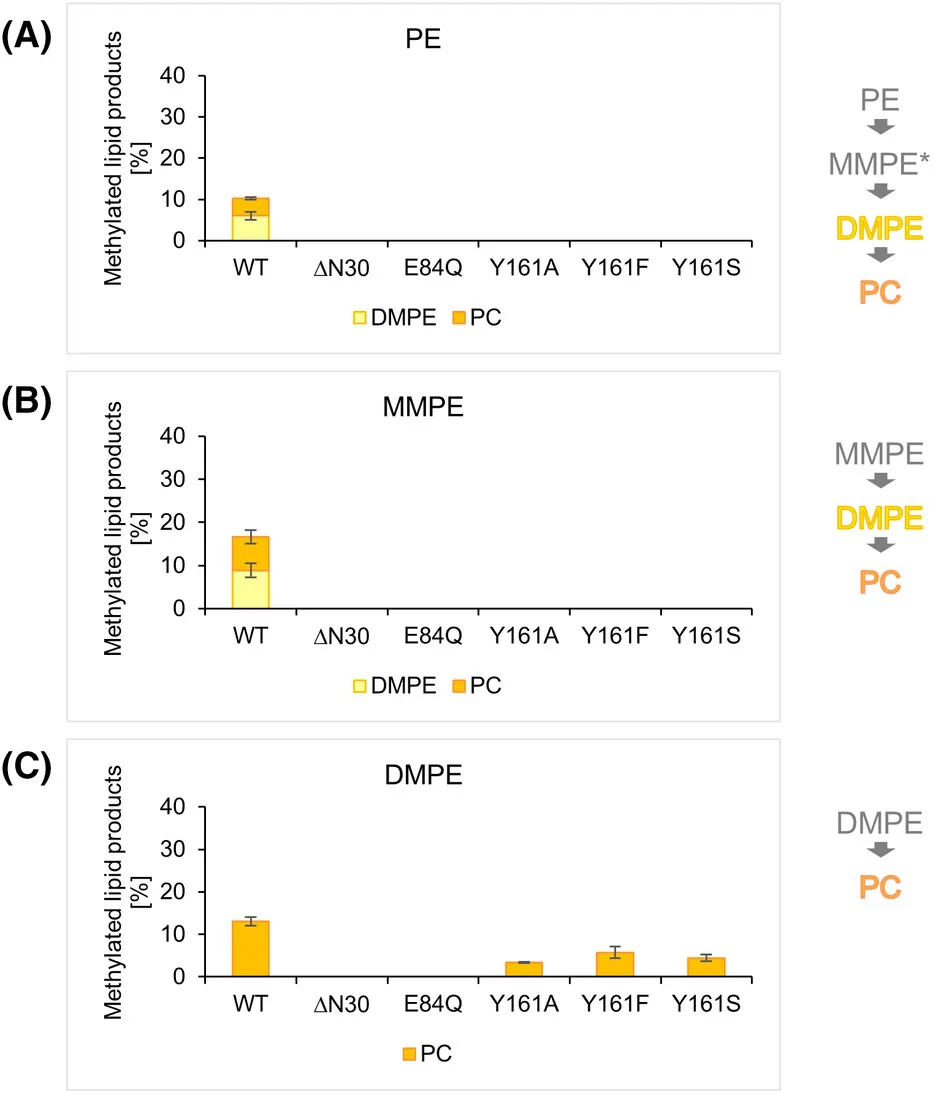
*In vitro* activity analysis of *At*PmtA upon addition of phosphatidylethanolamine (PE) (A), monomethyl‐PE (MMPE) (B), and dimethyl‐PE (DMPE) (C) in the presence of PG after 4 h at 30 °C. The total lipid content of each sample was normalized to 100%, and the relative amounts of formed products of the methylation pathway were assessed by measuring band intensity in the imagej software. MMPE* was left out of quantification because of its retention behavior. The data shown represent the mean values of two or more independent measurements. Error bars indicate standard deviation.

### Secondary structure analysis of *At*PmtA variants with and without PG liposomes

Secondary structure analysis of *At*PmtA and its variants revealed that all full‐length proteins were properly folded, with CD spectra comparable to that of the WT protein (Figs 8A and S8B). As expected, the *At*PmtA ∆N30, which lacks the N‐terminal αA‐helix, exhibited a reduced α‐helical content (Table S6, [47, 48, 49]).

**Fig. 8:**
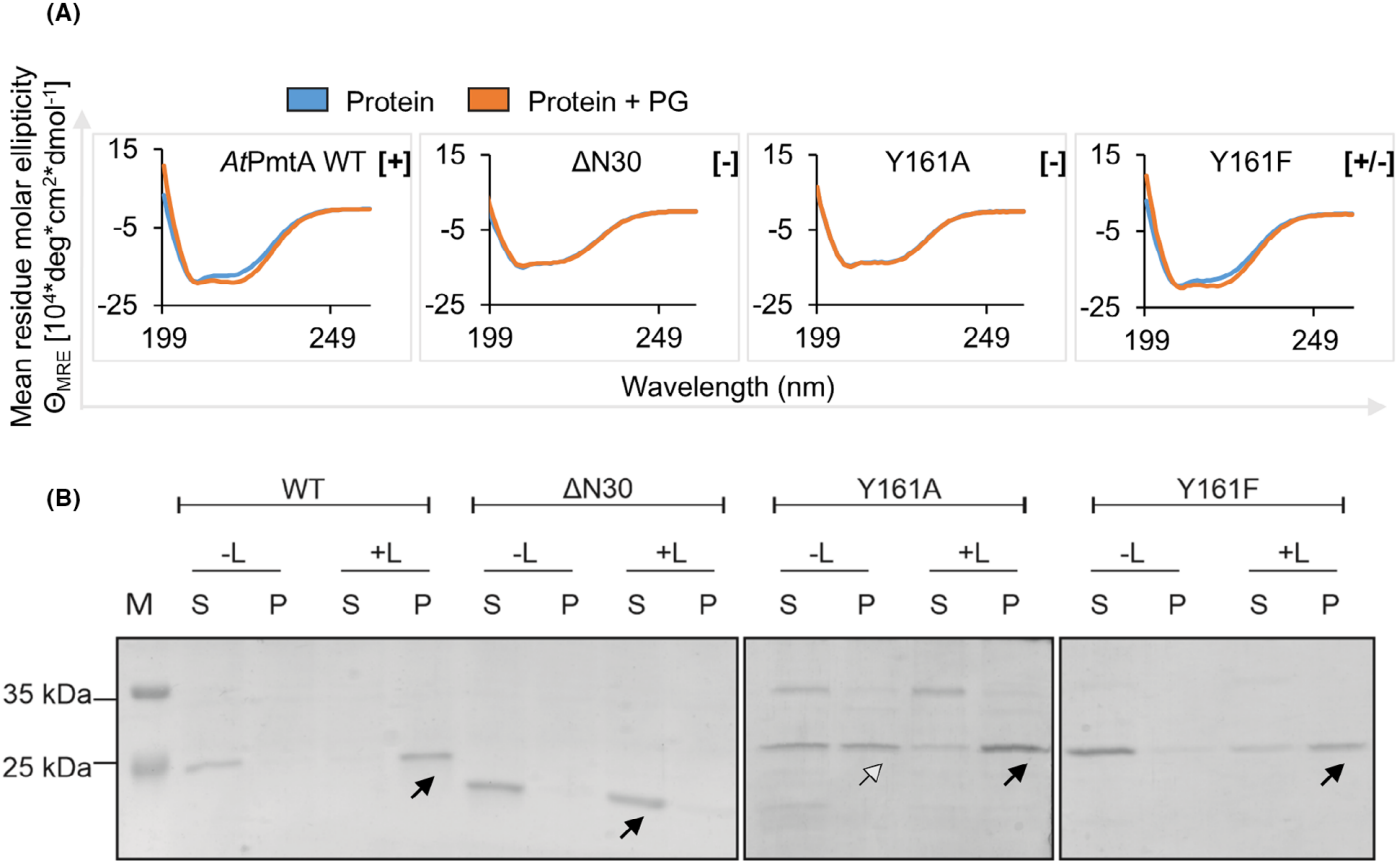
Circular dichroism (CD) spectroscopy analysis of *At*PmtA protein variants before and after incubation with phosphatidylglycerol (PG)‐consisting liposomes (A) and co‐sedimentation studies with liposomes composed of phosphatidylethanolamine (PE), PG and CL (B). A truncated variant of *At*PmtA ∆N30 served as a negative control. Filled arrows indicate protein behavior in the presence of liposomes; unfilled arrows indicate behavior in the absence of liposomes. The data shown represent the averaged spectra of two or more independent measurements. *In vivo* activity levels: [+]: WT‐like, [+/−]: reduced, [−]: inactive.

### Lipid binding of *At*PmtA variants

In contrast to *Rt*PmtA (Fig. 5A), the addition of PG‐containing liposomes induced only subtle changes in the CD spectrum of *At*PmtA (Figs 8A and S8B). As expected, the ∆N30 variant, lacking the N‐terminal helix required for membrane association, showed no spectral shift. Similarly, the inactive variants E84Q, Y161A, and Y161S also exhibited no detectable response to liposome addition (Figs 8A and S8B). Notably, BeStSel™ analysis revealed that Y161A and Y161S had a reduced α‐helical content, comparable to the ∆N30 variant (Table S6, [47, 48, 49]), which may account for their impaired membrane‐binding capability. In contrast, the catalytically active Y161F variant displayed a CD spectral shift like the WT protein, indicating preserved lipid‐induced conformational flexibility.

Liposome co‐sedimentation assays confirmed these results. In the absence of liposomes, WT *At*PmtA was detected exclusively in the supernatant but shifted to the pellet fraction in the presence of liposomes (indicated by a filled arrow in Fig. 8B). The ∆N30 variant, used as a negative control, remained in the supernatant fraction irrespective of liposome addition.

The Y161A variant was difficult to purify and showed a tendency to precipitate. Accordingly, approximately equal amounts of the protein were detected in the supernatant and pellet fractions even in the absence of liposomes (unfilled arrow in Fig. 8B). In the presence of liposomes, most of the protein shifted to the pellet fraction (filled arrow in Fig. 8B), indicating membrane binding. This interpretation is supported by the observation that a co‐purifying higher‐molecular‐mass protein remained in the supernatant regardless of the absence or presence of liposomes. The Y161F variant displayed WT‐like behavior, shifting predominantly to the pellet fraction when liposomes were present.

## Discussion

Phospholipid *N*‐methyltransferases are key enzymes in bacterial PC biosynthesis. Despite sharing less than 16% sequence identity, *A. tumefaciens* PmtA and *R. thermophilum* PmtA, representing two distinct Pmt classes, are both able to catalyze all three methylation steps of the pathway [10, 20].

Previous studies on various methyltransferases have emphasized the importance of specific amino acid residues, particularly tyrosines, as well as positively and negatively charged amino acids, in catalysis. For example, a critical tyrosine residue was identified in CbiL, a methyltransferase involved in anaerobic vitamin B12 biosynthesis in *Chlorobium tepidum*. In the *Plasmodium falciparum* phosphoethanolamine methyltransferase, both tyrosine and histidine were shown to be essential for activity [50, 51]. In NirE, a SAM‐dependent uroporphyrinogen III methyltransferase from *Pseudomonas aeruginosa*, a functional interplay between arginine and glutamine was found to be critical for enzymatic function [33].

In this study, we selected seven amino acid residues in the *R*‐type enzyme and three in the *S*‐type Pmt enzyme as candidate residues potentially important for catalytic activity. These amino acids were both highly conserved within their respective Pmt class and localized in the predicted ligand‐binding pockets. As negative controls, we included Y63 in *Rt*PmtA and Y85 in *At*PmtA that did not meet these criteria. As expected, alanine, phenylalanine, or serine substitutions at these control sites had no significant effect on enzymatic activity in *E. coli*, reinforcing the importance for both evolutionary conservation and spatial positioning in identifying catalytically relevant residues.

Furthermore, our *in vivo* activity assays ruled out the involvement of aspartate residues D18 (*Rt*PmtA), D99 (*Rt*PmtA), and D106 (*At*PmtA) in catalysis. The latter two residues had previously been proposed to coordinate the adenine moiety of the SAM molecule [13, 14]. D18 is located within the predicted substrate‐binding pocket in the AlphaFold 3 model of *Rt*PmtA. but not in the crystal structure, highlighting one of the few discrepancies between the predicted and the experimentally determined structure (Fig. S3, [13, 40, 41]).

### Essential residues in *Rt*PmtA

Based on our computational and biochemical analyses, we identified two tyrosines (Y10 and Y17) as key contributors to the catalytic mechanism of methyl group transfer in *Rt*PmtA. Substituting either residue significantly impacted enzyme activity: Y10A, Y10F, and Y10S mutants exhibited reduced activity, while Y17A and Y17S substitutions completely abolished activity. Phenylalanine substitutions were generally better tolerated than alanine and serine substitutions, suggesting that both the aromatic character and side‐chain size at these positions are critical for enzymatic function. Because replacement of both tyrosines in the double‐mutant Y10F/Y17F abolished *in vivo* activity, at least one of them appears to be required for enzymatic function. Notably, all tyrosine variants with reduced or abolished activity retained membrane‐binding capability, indicating that the loss of function results from impaired catalysis rather than defective membrane association.

Regarding the role of charged residues in catalysis, substitution of H117 with alanine markedly reduced or abolished both *in vivo* and *in vitro* activity. In contrast, H117F retained WT‐like activity. Given its bulky side chain, H117 has been proposed to regulate substrate access into the active site as a gatekeeper [13], supporting our findings on the importance of the side‐chain size at this position. Consistent with the interpretation, the equivalent position in other *R*‐type Pmts is typically occupied by residues with large aromatic ring structures, such as tyrosine or phenylalanine, suggesting a primarily structural rather than catalytic role (Fig. S1, [13, 40, 41]).

Another conserved histidine, H147, located in the predicted ligand‐binding pocket, was found to be similarly critical. Both H147A and H147F variants exhibited severely impaired production of methylated products *in vivo* and *in vitro*, underscoring its hypothesized function in phosphate group stabilization of DMPE through ionic interactions [13], a mechanism that may extend to other lipid substrates as well.

Substitution of D73, previously proposed to coordinate the ribose segment of SAM [13], resulted in a complete loss of enzymatic activity *in vivo* and only residual activity *in vitro*. However, CD spectroscopy revealed that the secondary structure of this variant differed from that of the WT protein spectrum, suggesting a structural rather than a direct catalytic role for D73. This interpretation is further supported by its spatial distance from the catalytically relevant residues Y10 and Y17, as well as the amine group of the lipid substrate (8.5–10.4 Å).

The substitution of E160 with glutamine resulted in only a minor decrease in enzyme activity. Given its considerable distance from the tyrosines possibly involved in methyl group transfer (Y10: 15.5 Å, Y17: 17.3 Å) and from the lipid‐substrate head group (DMPE: 13.2 Å), a direct role in catalysis appears unlikely.

Hydrophobic residues W13 and F21 have been proposed to contribute to catalytic activity by creating a hydrophobic microenvironment in the active site [13]. The substitution of W13 to alanine led to a general reduction in enzymatic activity both *in vivo* and *in vitro*. In contrast, the F21A variant was unable to methylate PE but could convert MMPE and DMPE. Activity assays based on SAH formation indicated that F21A retained higher catalytic activity than W13A during the final methylation step from DMPE to PC [13]. Together, these findings suggest that W13 and F21 are important for maintaining enzymatic function, likely by providing a favorable hydrophobic environment in the active site. However, their direct involvement in methyl group transfer appears unlikely.

### Essential residues in *At*PmtA

The finding that Y161 in *At*PmtA is essential for enzymatic activity [14] was confirmed by our mutagenesis studies. The Y161A and Y161S variants were inactive *in vivo*, whereas the Y161F variants retained residual activity. The loss of activity observed for Y161A and Y161S may be attributable, at least in part, to impaired membrane association. These results underscore the importance of membrane binding for catalytic function, in agreement with previous findings by Danne *et al*. [11].

Among the highly conserved acidic residues, E84, like D73 in *Rt*PmtA, has been postulated to stabilize the ribose group of SAM [14]. Substitution with glutamine (E84Q) resulted in a complete loss of activity, consistent with observations by Watanabe *et al*. [14]. Despite retaining secondary structure, the E84Q variant failed to associate with liposomes, indicating the loss of function likely stems from impaired membrane interaction rather than a direct catalytic defect. Docking studies performed in YASARA [52] placed E84 approximately 10.3 Å from the DMPE head group and 13.7 Å from Y161, supporting a structural or stabilizing role for this residue rather than direct involvement in methyl group transfer (Fig. S10, [14, 52, 53]).

### Potential catalytic mechanisms of *R*‐ and *S*‐type Pmts

#### *Rt*PmtA as *R*‐type representative

Salsabila and Kim proposed that Y10 and G171 are catalytically active residues in *Rt*PmtA. According to their model, the carbonyl group of G171 lowers the pKa of Y10, facilitating the deprotonation of its hydroxyl group and activating it for methyl group transfer [13]. However, our findings suggest that, in addition to Y10, Y17 also plays a critical role in the catalytic mechanism. Variants at this position showed significantly impaired activity despite retaining membrane binding (Fig. 9). Interestingly, both Y10 and Y17 are highly conserved and located on the N‐terminal α‐helix of the *R*‐type Pmts. Surprisingly, all Y10 variants displayed similar activity, implying that the specific structure of its side chain, such as the hydroxyl group, aromatic ring, or side‐chain length, is not critical for catalytic function. Notably, the complete activity loss of the double‐mutant Y10F/Y17F strongly supports a critical and dual role for Y17 in catalysis. The hydroxyl group appears important for proton transfer, while the aromatic ring likely contributes to hydrophobic interactions. The Y17F variant, which retains the aromatic ring but lacks the hydroxyl group, showed only a minor reduction in activity, suggesting that hydrophobic interactions alone can partially support function. Consistent with this assumption is the finding that substitution with alanine completely abolished activity, likely due to the loss of both functional groups. Similarly, the Y17S variant, containing a short carbon chain and a hydroxyl group, was unable to compensate for the absence of an aromatic ring and was also inactive.

**Fig. 9:**
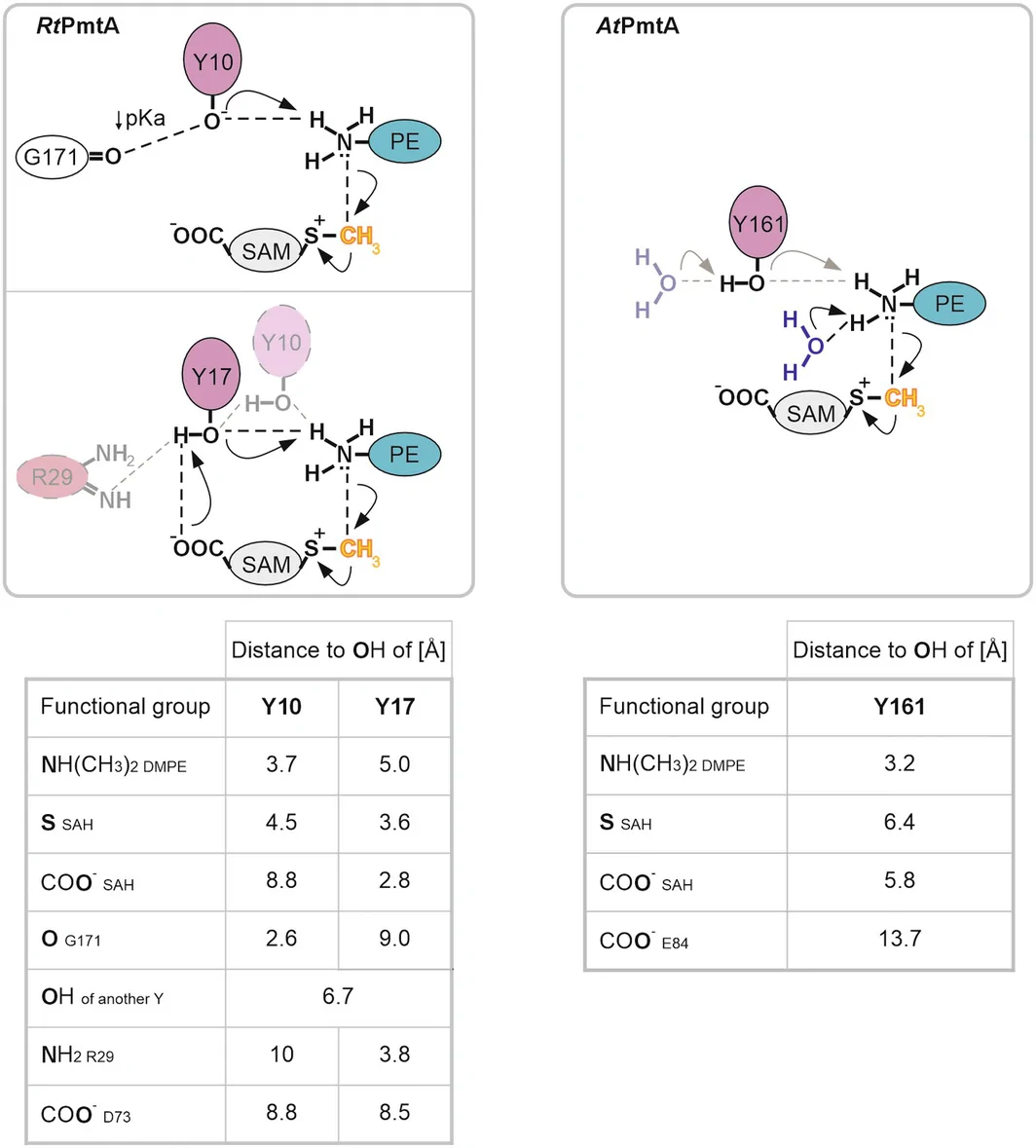
Proposed catalytic mechanisms of bacterial phospholipid *N*‐methyltransferases (Pmts). *Rt*PmtA: The catalytic mechanism proposed by Salsabila and Kim [13] for *Rt*PmtA involving Y10 and G171 residues is depicted on the upper left. The carbonyl group of G171 decreases the pKa of the Y10 hydroxyl group, activating it for substrate deprotonation followed by a nucleophilic attack on *S*‐adenosyl‐*l*‐methionine (SAM). On the lower left, the revised mechanism with Y17 as the key residue in methyl transfer catalysis is presented. Being activated by the carboxyl group of SAM and possibly kept in the active state by the located nearby R29, it deprotonates the lipid substrate alone or in combination with Y10. This is followed by a nucleophilic attack of the substrate on the methyl group of SAM. *At*PmtA: The potential catalytic mechanism of *At*PmtA mentioned by Watanabe *et al*. [14] and confirmed in our study is depicted on the right. Due to the absence of any charged residues possibly interacting with Y161 or the amine group of phosphatidylethanolamine (PE) in the putative binding pocket of this enzyme, *At*PmtA most likely relies on the proximity and desolvation mechanism. A solvating water molecule is proposed to directly deprotonate the amine group of a lipid substrate or activate Y161 for substrate deprotonation and further methyl group transfer. Distances in Å presented in the tables below were measured in the crystal structure with co‐crystallized dimethyl‐PE (DMPE) and SAH for *Rt*PmtA [13], as well as in the YASARA docking simulation with DMPE based on the crystal structure of *At*PmtA with co‐crystallized SAH (Fig. S10, [14, 52, 53]). The atoms selected for measurements are shown in bold.

Based on the orientation of Y17 in the crystal structure with the co‐crystallized SAH molecule, it was initially proposed that Y17 participates solely in stabilizing the carboxylate group of SAH [13]. However, its close proximity to the carboxyl group (OH_Y17_‐COO^−^_SAH_: 2.8 Å) of SAH suggests a potential for polarization of the tyrosine residue. Specifically, hydrogen bonding between the tyrosine hydroxyl group and the carboxyl group may increase the electrophilicity of the hydroxyl oxygen, rendering the oxygen more electron‐deficient and potentially more reactive. This enhanced polarization could facilitate a catalytic role for Y17, enabling interactions not only with the cofactor but also with other partners, such as Y10 (OH_Y17_‐OH_Y10_: 6.7 Å) and the amine group of the lipid substrates (OH_Y17_‐NH(CH_3_)_2 DMPE_: 5.0 Å) (Fig. 9). Together, these spatial relationships support a broader catalytic role for Y17 beyond simple SAH stabilization. Alternatively, the R29 residue, conserved in four of the six *R*‐type Pmts analyzed in this study and located just 3.8 Å away from Y17, may help stabilize the deprotonated form of Y17 or function analogously to the tyrosine‐histidine catalytic dyad observed in other methyltransferases [51, 54].

The potential involvement of the carboxyl group of SAM suggests the possibility of cofactor‐assisted catalysis in *Rt*PmtA. Some studies have proposed that negatively charged groups, such as the carboxylate of SAM, can act as general bases in enzymatic reactions, directly deprotonating substrate functional groups [55]. However, this scenario appears unlikely for *Rt*PmtA, as the distance between the carboxyl group of co‐crystallized SAH and the amine group of the lipid‐substrate DMPE is about 7.4 Å, a separation too large to support direct proton abstraction (Fig. 9). It is important to note, however, that these distance measurements were from the crystal structure of the enzyme in its postreaction state, with SAH and DMPE bound. Given that significant conformational changes are expected (Fig. 1) and observed upon PG binding alone (Figs 5A and S8A), it is plausible that the spatial arrangement shifts during the reaction. Therefore, the actual distances and interactions under physiological conditions, particularly in the presence of SAM and PE, may differ from those captured in the crystal.

#### *At*PmtA as *S*‐type representative

In the study by Watanabe *et al*. [14], *At*PmtA was proposed to rely on a single tyrosine residue, Y161, for its catalytic function. Our findings support this conclusion. First, we identified only one highly conserved tyrosine among the *S*‐type Pmts analyzed. In contrast to the two catalytically relevant tyrosine residues in *Rt*PmtA, Y161 is located deep in the protein core. Second, mutational analysis demonstrated the substitution of Y161 with either alanine or serine abolished enzyme activity, whereas replacement with phenylalanine retained residual function, paralleling the behavior observed for Y17 variants in *Rt*PmtA. Further support for a catalytic role of Y161 comes from docking analyses performed in YASARA [52], which positions Y161 just 3.2 Å from the amine group of the lipid substrate (Fig. S10, [14, 52, 53]). By contrast, the carboxyl group of co‐crystallized SAH was 5.8 Å away, making direct polarization of the tyrosine unlikely. Therefore, it is plausible that substrate deprotonation in *At*PmtA is facilitated not by cofactor assisted catalysis, but by a combination of water molecules within the active site, the surrounding pocket architecture and substantial conformational rearrangements upon substrate and cofactor binding (Fig. 9). A similar reaction mechanism has been proposed for the methyltransferase DnrK from *Streptomyces peucetius* [32].

## Materials and methods

### Materials

The phospholipids 1,2‐dioleoyl‐*sn*‐glycero‐3‐phosphoethanolamine (#850725), 1,2‐dioleoyl‐*sn*‐glycero‐3‐phosphoethanolamine‐*n*‐methyl (#850850), 1,2‐dioleoyl‐*sn*‐glycero‐3‐phosphoethanolamine‐*n*, *n*‐dimethyl (#850852), 1,2‐dioleoyl‐*sn*‐glycero‐3‐phosphocholine (#850375), 1,2‐dioleoyl‐*sn*‐glycero‐3‐phospho‐[1′‐rac‐glycerol] (sodium salt) (#840475), and *S*‐(5′‐adenosyl)‐*l*‐methionine *p*‐toluenesulfonate salt (#A2408) were obtained from Merck, Germany.

### Bioinformatic analysis

#### Identification of amino acids important for catalytic activity

Amino acid sequence alignments were performed groupwise (Figs S1 [13, 40, 41] and S2, [11, 14, 40, 41]) using clustal omega [40]. The structural analysis was conducted in order to identify highly conserved amino acids in the putative binding pocket and investigate their effect on the catalytic activity of *Rt*PmtA and *At*PmtA. For that, protein sequences of *Rt*PmtA and *At*PmtA were uploaded to AlphaFold 3 [53] to generate protein structure models for subsequent bioinformatics investigation. The models were then evaluated using the P2Rank online service for pocket prediction [41, 42, 43] to elucidate potential lipid‐substrate‐binding sites. The crystal structures of *Rt*PmtA and *At*PmtA that became available during the course of our study were examined using the same bioinformatic tools and compared with the predicted models (Figs, S3 [13, 40, 41] and S4, [14, 40, 41]).

#### *In silico* substrate docking

Substrate docking simulation was performed using autodock VINA [56] within the YASARA molecular modeling program [52], utilizing *At*PmtA ∆25‐SAH structure [14]. The pre‐defined simulation cell (5×5×5 Å) was positioned within the potential lipid‐binding site close to the SAM‐binding motif. This site was derived by combining P2Rank with comparative structural analysis of the *Rt*PmtA structure [13], co‐crystallized SAH and DMPE. The *At*PmtA ∆25‐SAH complex was treated as rigid, while all ligands' internal degrees of freedom were considered. All other VINA parameters were kept at their default settings.

### Plasmid construction

The overview of used bacterial strains and created constructs in this study is presented in Table S7, [10, 11, 20]. Protein variants were created using pET28b vector as an overexpression system with the Q5^®^ Site‐Directed Mutagenesis Kit (no.: E0554S; NEB, Ipswich, MA, USA) and oligonucleotides listed in Table S8, following the manufacturer's instructions. This vector features a kanamycin resistance cassette and allows for inducible gene expression by isopropyl‐*β*‐*d*‐thiogalactopyranoside (IPTG). Additionally, it encodes an N‐terminal His‐tag for affinity purification.

### Heterologous production and *in vivo* activity test in *Escherichia coli*

The resulting expression plasmids for production of WT and mutated protein variants were transformed into *Escherichia coli* NiCo21 (DE3) cells. NiCo21 cells carrying the pET28b expression plasmids were cultured at 37 °C in LB broth with 50 μg·mL^−1^ kanamycin to an optical density of 0.6 at 600 nm (OD_600_). After inducing protein production by the addition of 1 mm IPTG, cultures were incubated at 30 °C for 4 h. Cells were harvested by centrifugation at the indicated time points and utilized to confirm recombinant protein production by SDS/polyacrylamide gel electrophoresis (PAGE) and western blot. Cell pellets were diluted in Laemmli sample buffer (0.31 m Tris base, 0.35 m SDS, 7.23 mm bromophenol blue, 50% glycerol, 25% 2‐mercapto‐ethanol, pH 6.8 adjusted with HCl), incubated at 95 °C for 10 min, and subsequently separated by SDS/PAGE using 12.5% gels. The gels then were stained with Coomassie Blue (0.1% Coomassie Blue R250 in 10% acetic acid, 50% methanol, and 40% H_2_O). For western blot analysis, proteins were transferred to the nitrocellulose blotting membrane (Amersham™ Protran™, Cytiva, Marlborough, MA, USA) and detected using the His‐Tag antibody HRP conjugate (Qiagen, Venlo, the Netherlands) according to manufacturer's instructions. For activity testing of the heterologously produced proteins, the OD_600_ of the cultures was adjusted to 3, and 1 mL of each culture was used for lipid analysis.

### Overexpression and purification of protein variants

For protein overexpression, similar to *in vivo* activity testing, NiCo21 cells transformed with the respective plasmid for different protein variants were utilized. NiCo21 cells were cultured at 37 °C in terrific broth (TB) with 50 μg·mL^−1^ kanamycin to OD_600_ of 0.6–0.8. Cultures were rested for 30 min on ice prior to induction with 0.1 mm IPTG followed by incubation at 18 °C for 18 h.

Proteins were purified according to established protocols for *At*PmtA with minor modifications [10]. Briefly, 1 L of *E. coli* expression culture was pelleted. Cells were lysed with the addition of cOmplete™ EDTA‐free protease inhibitor cocktail (Roche, Basel, Switzerland) using B‐PER™ Complete bacterial protein extraction reagent (Thermo Fisher Scientific, Waltham, MA, USA) according to the manufacturer's instructions. Cell debris was removed from the soluble fraction by centrifugation (30 600 ***g***, 30 min, 4 °C). The resulting supernatant was then applied to nickel‐iminodiacetic acid columns (Protino Ni‐IDA 2000 packed columns; Macherey‐Nagel, Düren, Germany). Nonspecifically bound proteins were removed by washing the column with 10 column volumes (cv) of LEW buffer (50 mm NaH_2_PO_4_, 300 mm NaCl, pH 8.0 adjusted with NaOH) containing 10 mm imidazole, 5 cv LEW buffer containing 30 mm imidazole, and 5 cv LEW buffer containing 50 mm imidazole. The His‐tagged protein variants were then eluted using LEW buffer containing 500 mm imidazole. Wash and elution fractions from the column were collected, diluted in Laemmli buffer and subjected to analysis by SDS/PAGE and Coomassie Blue staining to ascertain protein purification profile. For further studies, eluted proteins were concentrated using centrifugal filter units (10 kDa cutoff, Amicon Ultra‐15; Merck Millipore, Billerica, MA, USA) before (*At*PmtA variants) or after (*Rt*PmtA variants) buffer exchange in PD‐10 columns against low LEW buffer (50 mm NaH_2_PO_4_, 50 mm NaCl, pH 8.0 adjusted with NaOH) with lower salt concentration without imidazole. Final protein concentrations were measured using the Pierce BCA protein assay kit (Thermo Fisher Scientific, USA) according to the manufacturer's instructions.

### *In vitro* activity test

Experimental conditions for *in vitro* activity tests were adapted from protocols for *At*PmtA [10]. The activity of purified Pmts was assessed in a total volume of 100 μL containing 0.4 μm protein, 1 mm SAM, 0.65 mm lipid substrate (PE/MMPE/DMPE), and, optionally, 0.63 mm anionic lipid (PG) in low LEW buffer with 0.05% Triton X‐100. Enzymatic activity was tested for 4 h at 30 °C under gentle shaking. After the reactions were stopped by addition of 375 μL methanol/chloroform (2 : 1) mixture, lipids were isolated and analyzed as described in the section below.

### Lipid analysis

Lipids were extracted as described by Bligh and Dyer (57) and then separated by thin‐layer chromatography (TLC) using HPTLC silica gel 60 plates (Merck; USA). TLC was carried out with *n*‐propanol/propionic acid/chloroform/distilled water (3 : 2 : 2 : 1) as the mobile phase. Copper sulfate staining (300 mm CuSO_4_ × 5 H_2_O, 8.5% [vol/vol] phosphoric acid) was employed to nonspecifically visualize separated lipids at 170 °C for 10 min. Lipid band intensity was measured in imagej [44] and used to assess protein activity *in vivo* and *in vitro*. The total lipid amount of each sample was normalized to 100%, and only the relative levels of the intermediate products DMPE and PC were determined. The amount of MMPE could not always be identified due to its overlap with PE, PG, and CL in the lipid cloud, all of which had the same retention behavior.

### Circular dichroism spectroscopy

#### Secondary structure analysis

To assess whether the proteins of interest were properly folded, purified samples were analyzed using circular dichroism (CD) spectroscopy. CD spectra of purified proteins were recorded at least twice between 190 and 260 nm, bandwidth 1 nm, using Chirascan™ circular dichroism spectrometer (Applied Photophysics Ltd.; Leatherhead, Surrey, UK) at 20 °C in low LEW buffer. The samples contained 0.1 mg·mL^−1^ of recombinant protein.

#### Membrane‐binding studies by CD spectroscopy

CD spectroscopy was also used to assess the membrane‐binding ability of the proteins using PG liposomes as a model membrane. A shift in the spectrum upon liposome addition indicated membrane binding. For liposome preparation, corresponding PG amounts dissolved in chloroform/methanol (1 : 1) were dried in a vacuum centrifuge for 30 min to form a lipid film and hydrated in low LEW buffer by vortexing for 1 h. To achieve uniform size distribution, the resultant liposomes were subsequently extruded (Avanti Mini; Avanti Polar Lipids, no.: 610000, Sweden) through a polycarbonate membrane of 0.4 μm in diameter. Prepared liposomes were stored on ice and used on the same day. To assess protein‐membrane interaction, 0.2 mm liposomes were pre‐incubated with 0.1 mg·mL^−1^ protein at room temperature for 20 min. CD spectra were recorded as described above.

### Membrane‐binding studies using liposome co‐sedimentation assay

To further evaluate membrane‐binding properties of selected inactive protein variants and confirm the findings from the CD spectroscopic analysis, liposome co‐sedimentation assays were conducted. Liposomes consisting of PE, PG, and CL in a ratio of 7 : 2 : 1 (mol%) were used, representing the typical composition of bacterial membranes. The liposomes were prepared as described above for the CD spectroscopy. To assess the binding of protein to liposomes, 7.5 μm protein was incubated with 0.5 mm liposomes at room temperature for 30 min, in a total volume of 150 μL. Subsequently, samples were filled up to 600 μL for ultracentrifugation at 4 °C at 300 000 ***g*** (Beckmann Optima™ MAX‐XP ultracentrifuge, rotor TLA 120.2, USA) for 30 min. The supernatant and pellet fractions were collected and adjusted to equal volumes for comparison and analyzed via SDS/PAGE, with 20 μL of each fraction loaded onto the gel.

## Author contributions

IS, MA, and FN conceptualized and supervised the experiments. IS, LSF, LMK, and AE performed the experiments. FN provided infrastructure. IS and LSF analyzed and validated the data. IS, MA, and FN wrote the manuscript. FN and MA supervised the project and FN acquired funding.

## Conflict of interest

The authors declare no conflict of interest.

## Supporting information


**Fig. S1.** Amino acid sequence alignment of *R*‐type Pmts performed in Clustal Omega.
**Fig. S2.** Amino acid sequence alignment of *S*‐type Pmts performed in Clustal Omega.
**Fig. S3.** Comparison of *Rt*PmtA crystal structure and its AlphaFold 3 model.
**Fig. S4.** Comparison of *At*PmtA crystal structure and its AlphaFold 3 model.
**Fig. S5.** SDS/PAGE and western blot analysis of PmtA protein overproduction in *E. coli* NiCo21 (DE) cultures.
**Fig. S6.** SDS/PAGE purification profiles of selected PmtA variants.
**Fig. S7.** Assessment of the *in vitro* activity of *Rt*PmtA and its variants using various substrates, both with and without PG.
**Fig. S8.** CD spectroscopy analysis of PmtA variants before and after incubation with PG‐consisting liposomes.
**Fig. S9.** *In vitro* activity assay of *At*PmtA and its variants with different substrates in the presence and absence of PG.
**Fig. S10.** Substrate docking of DMPE based on the crystal structure of *At*PmtA co‐crystallized with SAH.
**Table S1.** Membrane phospholipid composition of *E. coli* NiCo21 (DE3) expressing *Rt*PmtA variants.
**Table S2.** Quantification of methylated PE products from *in vitro* activity assays of *Rt*PmtA variants using PE, MMPE and DMPE as substrates in the presence of PG.
**Table S3.** Secondary structure analysis of *Rt*PmtA variants using BeStSel™ online tool.
**Table S4.** Membrane phospholipid composition of *E. coli* NiCo21 (DE3) expressing *At*PmtA variants.
**Table S5.** Quantification of methylated PE products from *in vitro* activity assays of *At*PmtA variants using PE, MMPE and DMPE as substrates in the presence of PG.
**Table S6.** Secondary structure analysis of *At*PmtA variants using BeStSel™ online tool.
**Table S7.** Strains and plasmid constructs used in this study.
**Table S8.** Oligonucleotides used in this study.

## Data Availability

The data that support the findings of this study are available in the Supporting Information of this article.
