# Supplementary material for: Toward understanding the catalytic mechanism for two classes of bacterial phospholipid N‐methyltransferases
Source: FEBS J. 2026 May 2;293(20):6253–71. doi: 10.1111/febs.70571 (PMC13669648; doi:10.1111/febs.70571)
Supplement: Supplementary file 1 [file FEBS-293-6253-s001.pdf]

Supporting Information

Towards understanding the catalytic mechanism for two classes of bacterial phospholipid *N*-methyltransferases

Irina Shevyreva, Lena Sophie Fritsch, Leander Mika Koch, Anton Effing, Meriyem Aktas and Franz Narberhaus*

Microbial Biology, Faculty of Biology and Biotechnology, Ruhr University Bochum, Bochum, Germany

*Address correspondence to Franz Narberhaus, Microbial Biology, Faculty of Biology and Biotechnology, Ruhr University Bochum, Universitätsstr. 150, 44780 Bochum, Germany

Tel: +49 (234) 32 23100

Franz.Narberhaus@ruhr-uni-bochum.de

This PDF file includes:

Figures S1 to S10

Tables S1 to S8

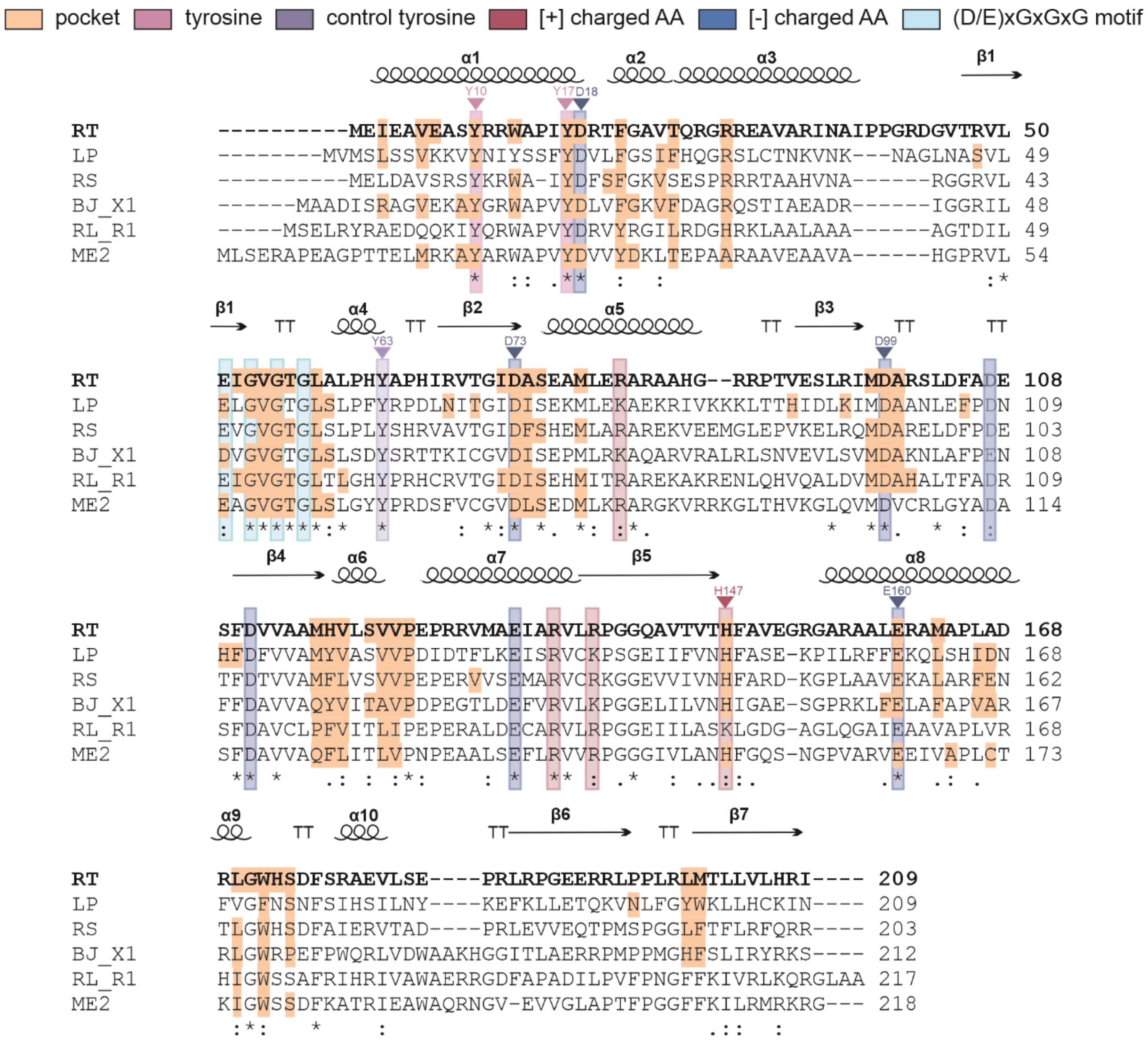

Figure S1. Amino acid sequence alignment of *R*-type Pmts performed in Clustal Omega. Amino acids predicted by P2Rank online tool to be in the putative pocket are labeled in orange [33,34]. Highly conserved amino acids of interest localized in the predicted pockets are tyrosines (pink), positively ([+], red) and negatively ([-], blue) charged amino acids. Control tyrosines (purple) were selected and included into analysis, as they were either highly conserved or present in the predicted pocket. The SAM-binding motif (D/E)xGxGxG is shown in light blue. RT: *Rubellimicrobium thermophilum* PmtA (Ruthe_01610), LP: *Legionella pneumophila* PmtA (WP_010947869), RS: *Rhodobacter sphaeroides* PmtA (Q05197)*,* BJ_X1: *Bradyrhizobium diazoefficiens* (formerly *B. japonicum*) PmtX1 (BAC52259)*,* RL_R1: *Rhizobium leguminosarum* PmtR1 (DLJ82_4359), ME2: *Methylorubrum extorquens* Pmt2 (ACS42118). The secondary structures are labelled according to Salsabila & Kim (2024) [14]: α with squiggles for alpha helices, β with arrows for β-strands, TT for turns.

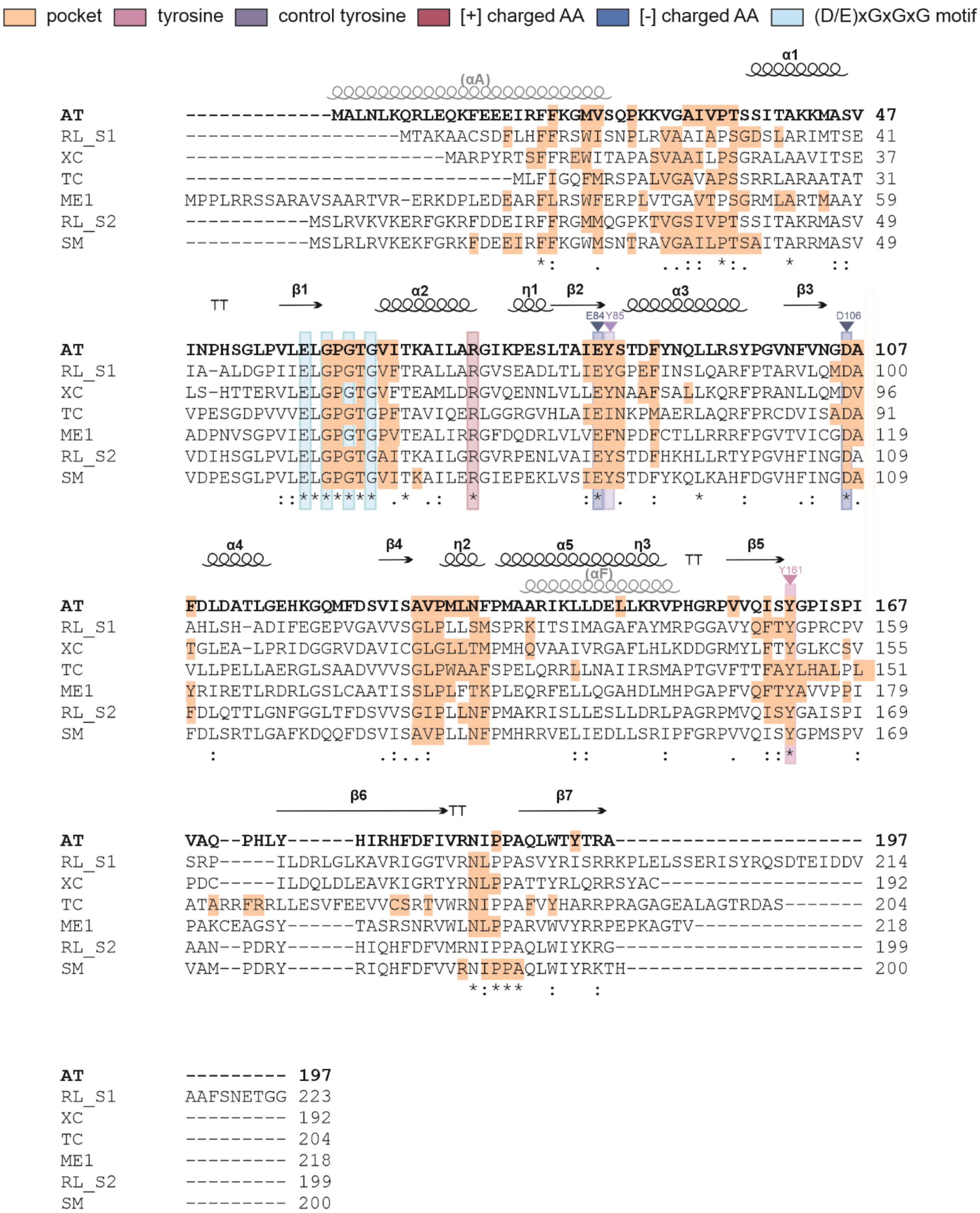

Figure S2. Amino acid sequence alignment of *S*-type Pmts performed in Clustal Omega. Amino acids predicted by P2Rank online tool to be in the putative pocket are labeled in orange [33,34]. Highly conserved amino acids of interest localized in the predicted pockets are tyrosines (pink), positively ([+], red) and negatively ([-], blue) charged amino acids. Control tyrosines (purple) were selected and included into analysis, as they were either highly conserved or present in the predicted pocket. The SAM-binding motif (D/E)xGxGxG is shown in light blue. AT: *Agrobacterium tumefaciens* PmtA (AAK86115), RL_S1: *R. leguminosarum* PmtS1 (DLJ82_3449), XC: *Xanthomonas campestris* PmtA (CAP49368), TC: *Thermostaphylospora chromogena* Pmt (SAMN04489764_3900), ME1: *M. extroquens* Pmt1 (ABY31351), RL_S2: *R. leguminosarum* PmtS2 (DLJ82_3306), SM: *Sinorhizobium meliloti* PmtA (Q92SN7). The secondary structures are labelled according to

Watanabe et al. (2025) [16] in black and Danne et al. (2015) [11]: α with squiggles for alpha helices, η with squiggles for 3_{10} helices, β with arrows for β-strands, TT for turns.

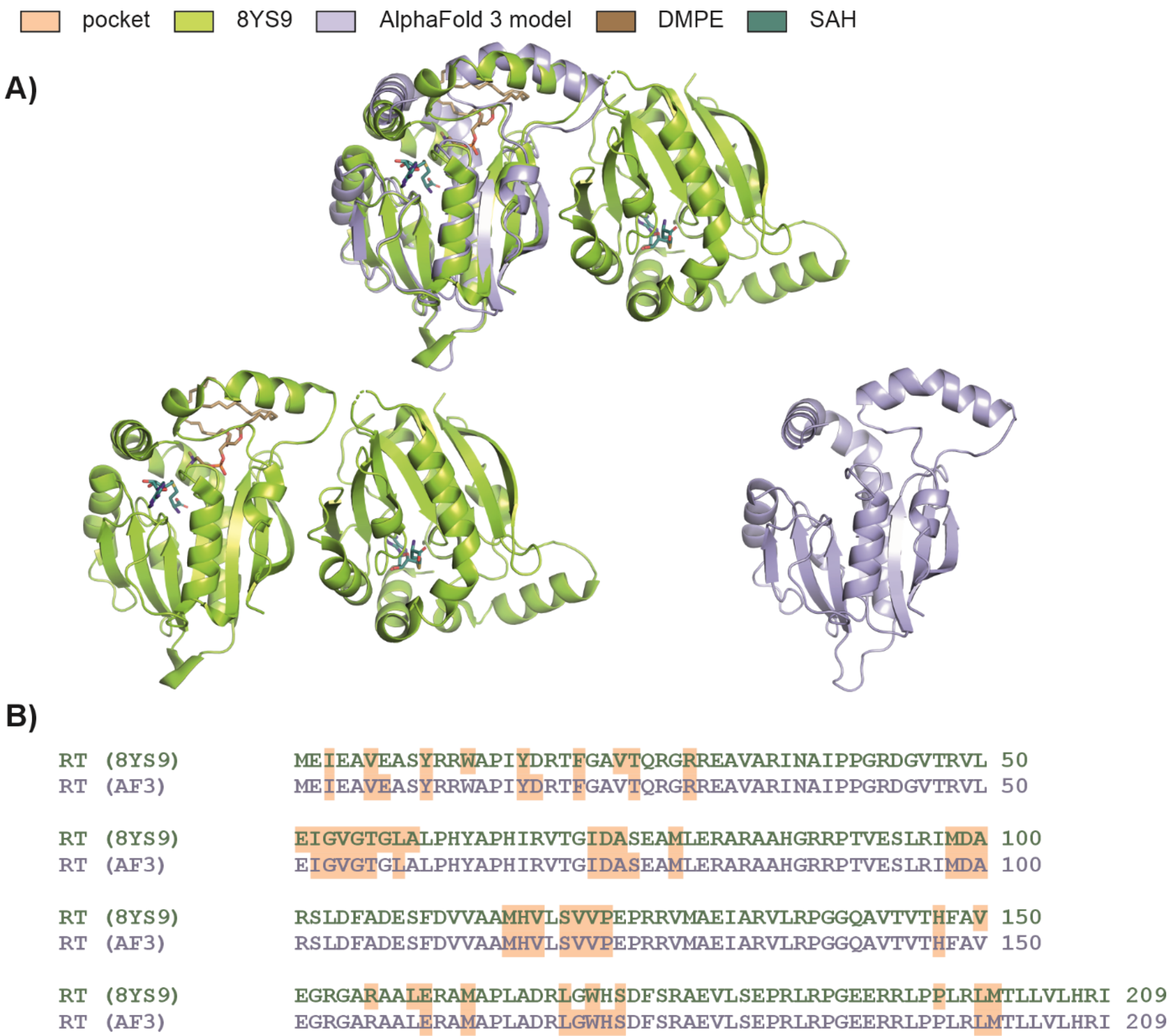

Figure S3. Comparison of *Rt*PmtA crystal structure and its AlphaFold 3 model. (A) Structural alignment of the *Rt*PmtA crystal structure (8YS9, light green, [14]) and the AlphaFold 3 model (light purple). (B) Mapping of predicted substrate-binding pockets (orange) onto the sequence alignment [33]. Crystal structure is comprised of two protein molecules co-crystallized with SAH and DMPE or SAH only. Substrate-binding pocket prediction was performed using P2Rank online tool [34]. DMPE: dimethylphosphatidylethanolamine, SAH: S-adenosylhomocysteine.

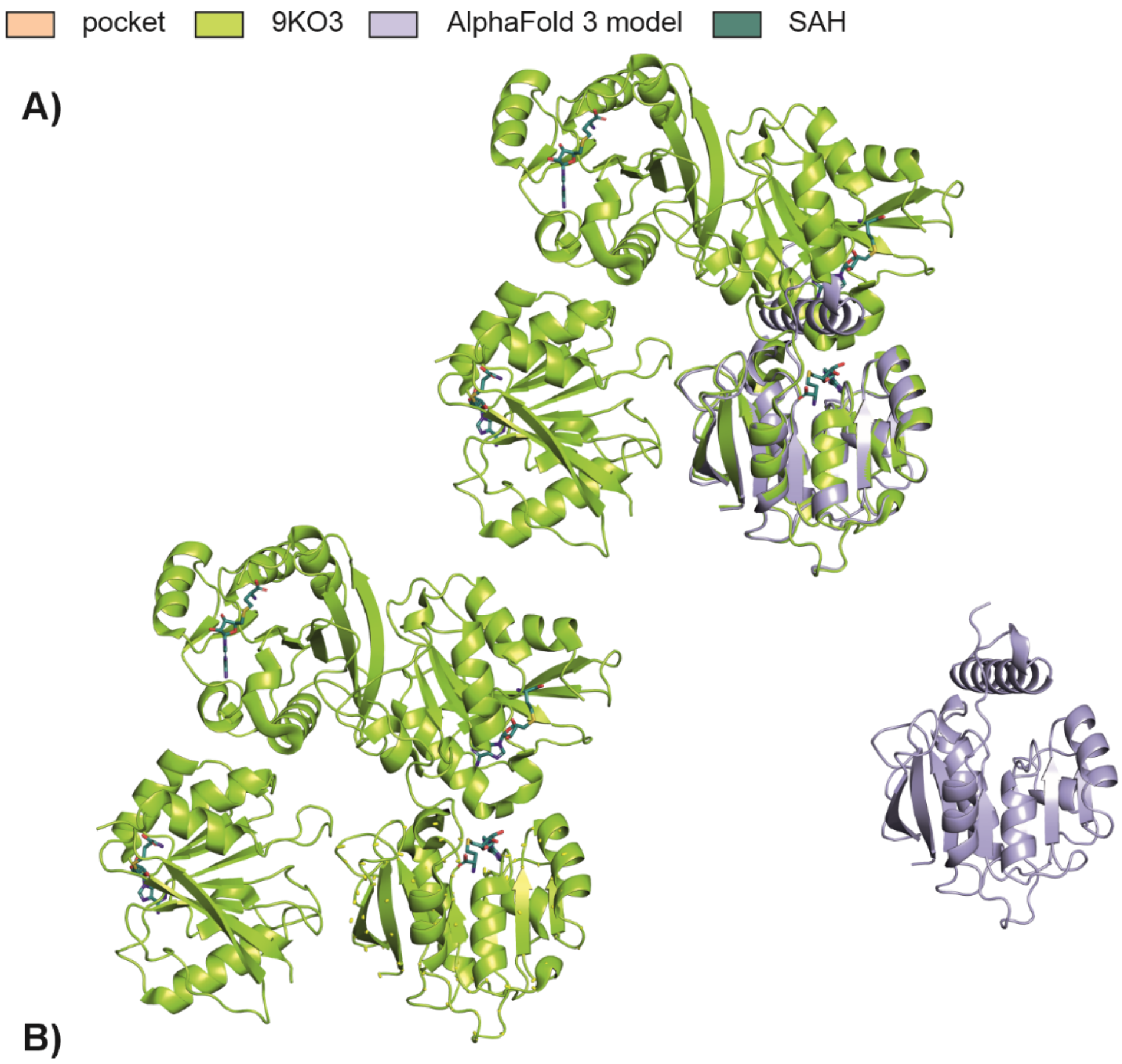

B)

```
AT (9KO3)     -------------------------QPKKVGAIVPTSSITAKKMASVINP 50
AT (AF3)      MALNLKQRLEQKFEEEIRFFKGMVSQPKKVGAIVPTSSITAKKMASVINP 50

AT (9KO3)     HSGLPVLELGPGTGVITKAILARGIKPESLTAIEYSTDFYNQLLRSYPGV 100
AT (AF3)      HSGLPVLELGPGTGVITKAILARGIKPESLTAIEYSTDFYNQLLRSYPGV 100

AT (9KO3)     NFVNGDAFDLDATLGEHKGQMFDSVISAVPMLNFPMAARIKLLDELLKRV 150
AT (AF3)      NFVNGDAFDLDATLGEHKGQMFDSVISAVPMLNFPMAARIKLLDELLKRV 150

AT (9KO3)     PHGRPVVQISYGPISPIVAQPHLYHIRHFDFIVRNIPPAQLWTYTRA 197
AT (AF3)      PHGRPVVQISYGPISPIVAQPHLYHIRHFDFIVRNIPPAQLWTYTRA 197
```

Figure S4. Comparison of *At*PmtA crystal structure and its AlphaFold 3 model. (A) Structural alignment of the AtPmtA crystal structure (9KO3, light green, [16]) and the AlphaFold 3 model (light purple). (B) Labeling of predicted substrate-binding pockets (orange) in the sequence alignment [33]. Crystal structure is comprised of four protein molecules co-crystallized with SAH. Substrate-binding pocket prediction was performed using P2Rank online tool [34].

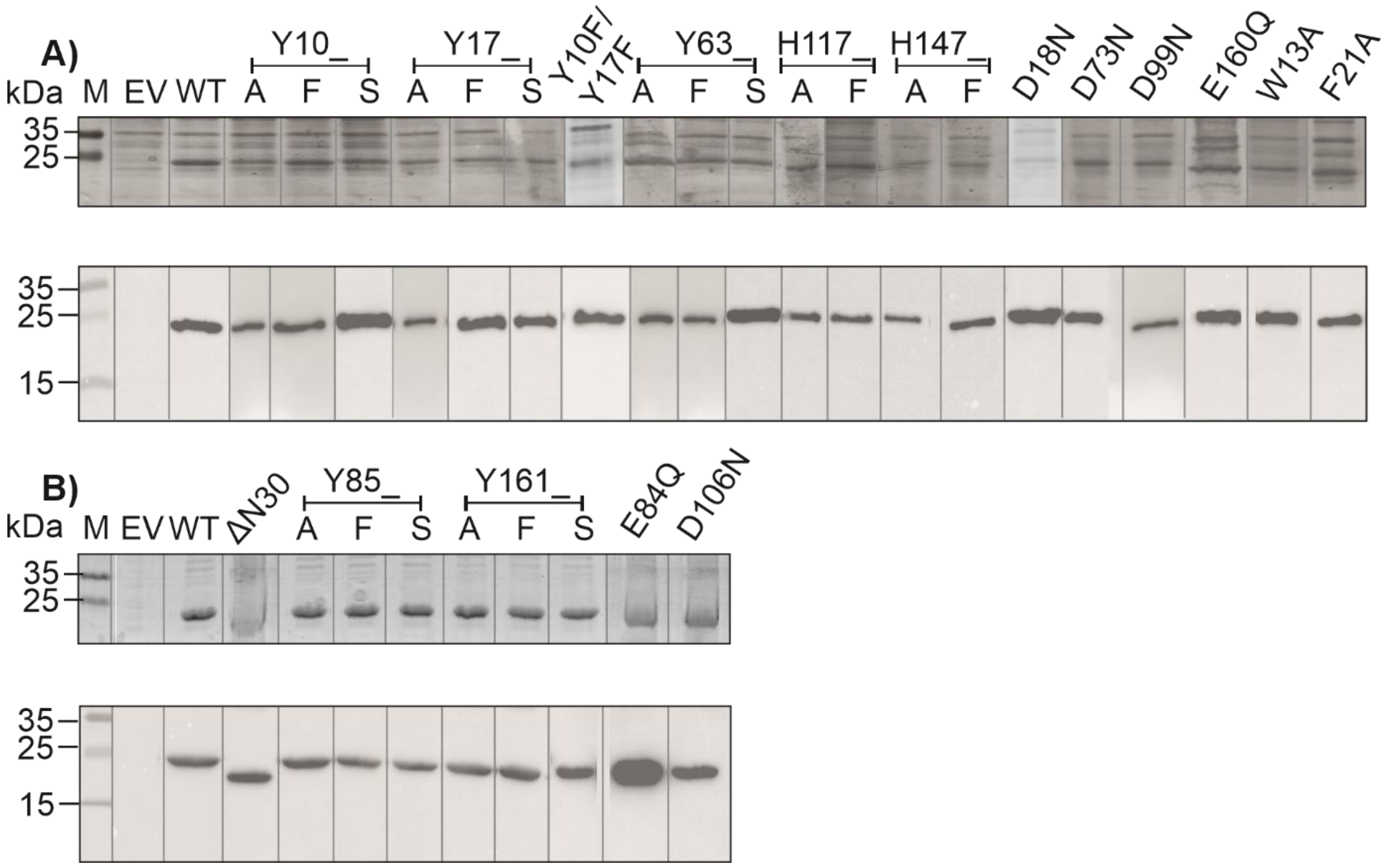

Figure S5. SDS-PAGE and Western blot analysis of PmtA protein overproduction in *E. coli* NiCo21 (DE) cultures. (A) *Rt*PmtA and (B) *At*PmtA, and their protein variants. The bands around 25 kDa for *Rt*PmtA and 24 kDa for *At*PmtA variants indicated successful overproduction after IPTG induction. SDS-PAGE gels were stained with Coomassie Blue staining. Images are displayed in grayscale. The SDS-PAGE gel is shown above, and the corresponding Western blot is displayed below.

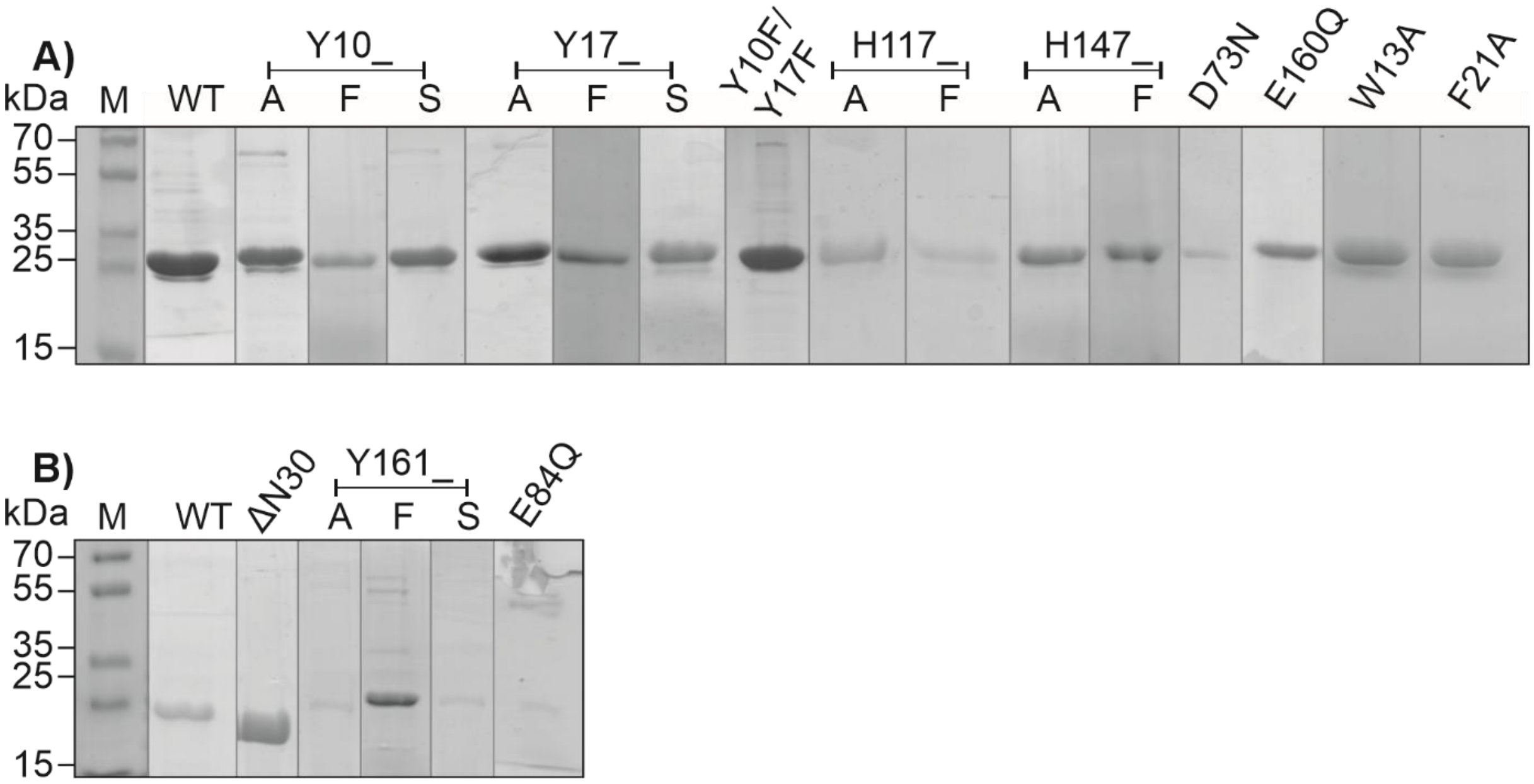

Figure S6. SDS-PAGE purification profiles of selected PmtA variants. (A) *Rt*PmtA and (B) *At*PmtA protein variants. Each protein variant's elution fraction was re-buffered and concentrated. Fifteen microliters of the resulting sample were loaded onto the SDS-PAGE gel. SDS-PAGE gels were stained with Coomassie Blue staining. Images are displayed in grayscale.

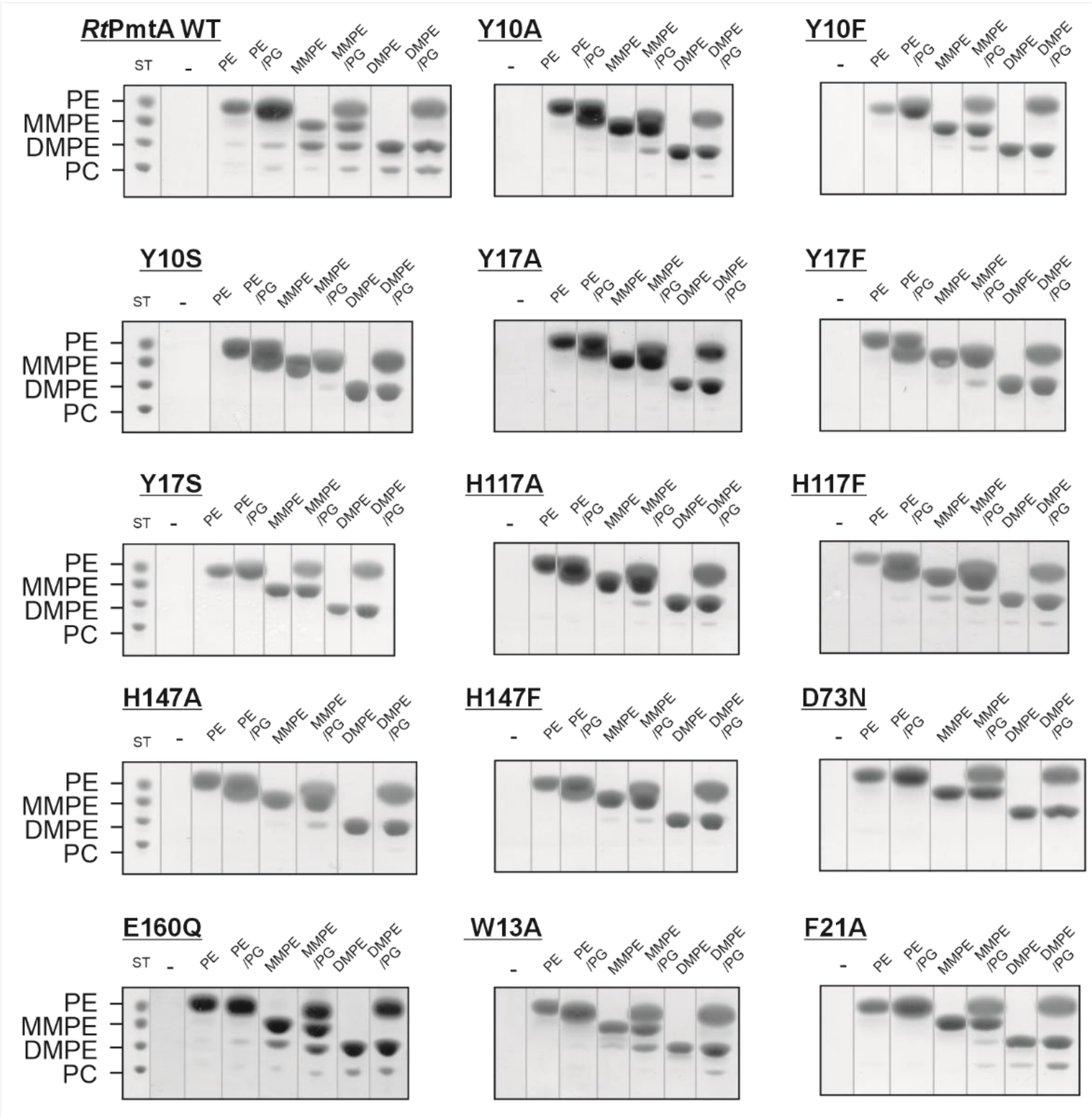

Figure S7. Assessment of the *in vitro* activity of *Rt*PmtA and its variants using various substrates, both with and without PG. Negative control (-) containing only protein and SAM was included in order to check for lipid co-purification in the initial protein sample. Relative amounts of formed products were quantified using ImageJ and presented in Figure 4 and Table S2. Lipid substrates used in the assay, PG or the lipid bands that could not be quantified due to co-migration with other lipids, such as MMPE, were classified as other lipids. TLC plates were stained with Copper sulfate staining. Images are shown in grayscale.

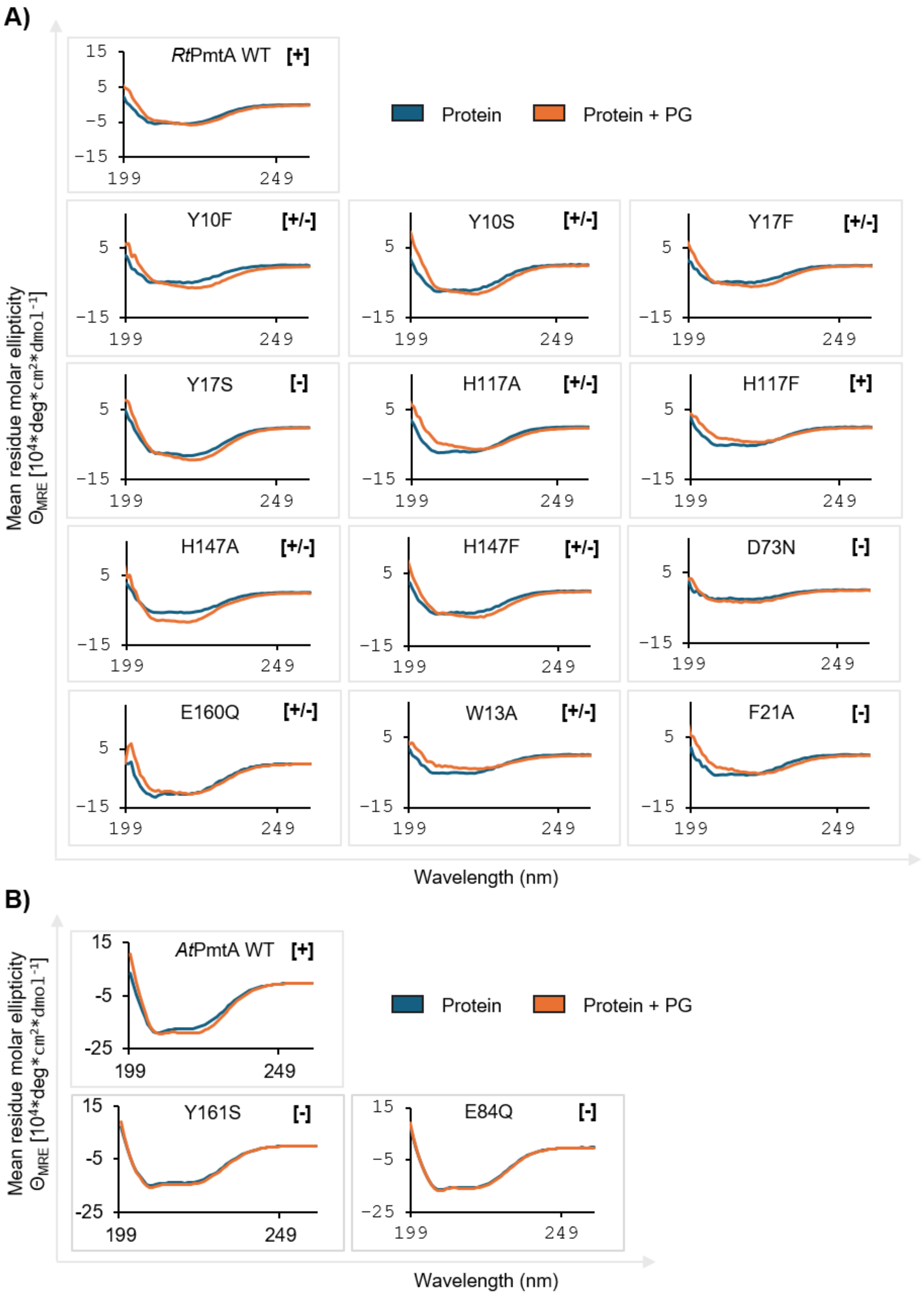

Figure S8. CD spectroscopy analysis of PmtA variants before and after incubation with PG-consisting liposomes. (A) *Rt*PmtA and (B) *At*PmtA protein variants. The measurements were performed between 190 and 260 nm at 20°C. The data shown represents the averaged spectra of two or more independent measurements. *In vivo* activity levels are indicated as follows: [+]: WT-like, [+/-]: reduced, [-]: inactive.

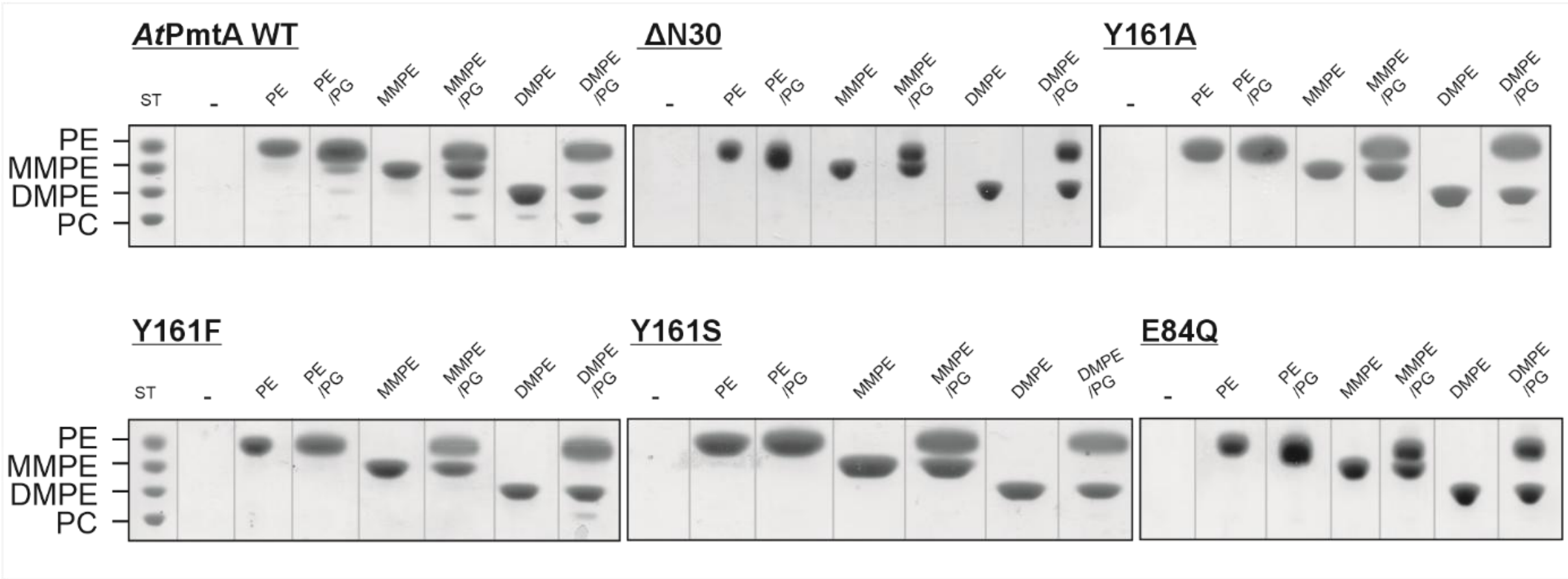

Figure S9. *In vitro* activity assay of *At*PmtA and its variants with different substrates in the presence and absence of PG. The experimental conditions and data analysis were the same as those described in Figure S7.

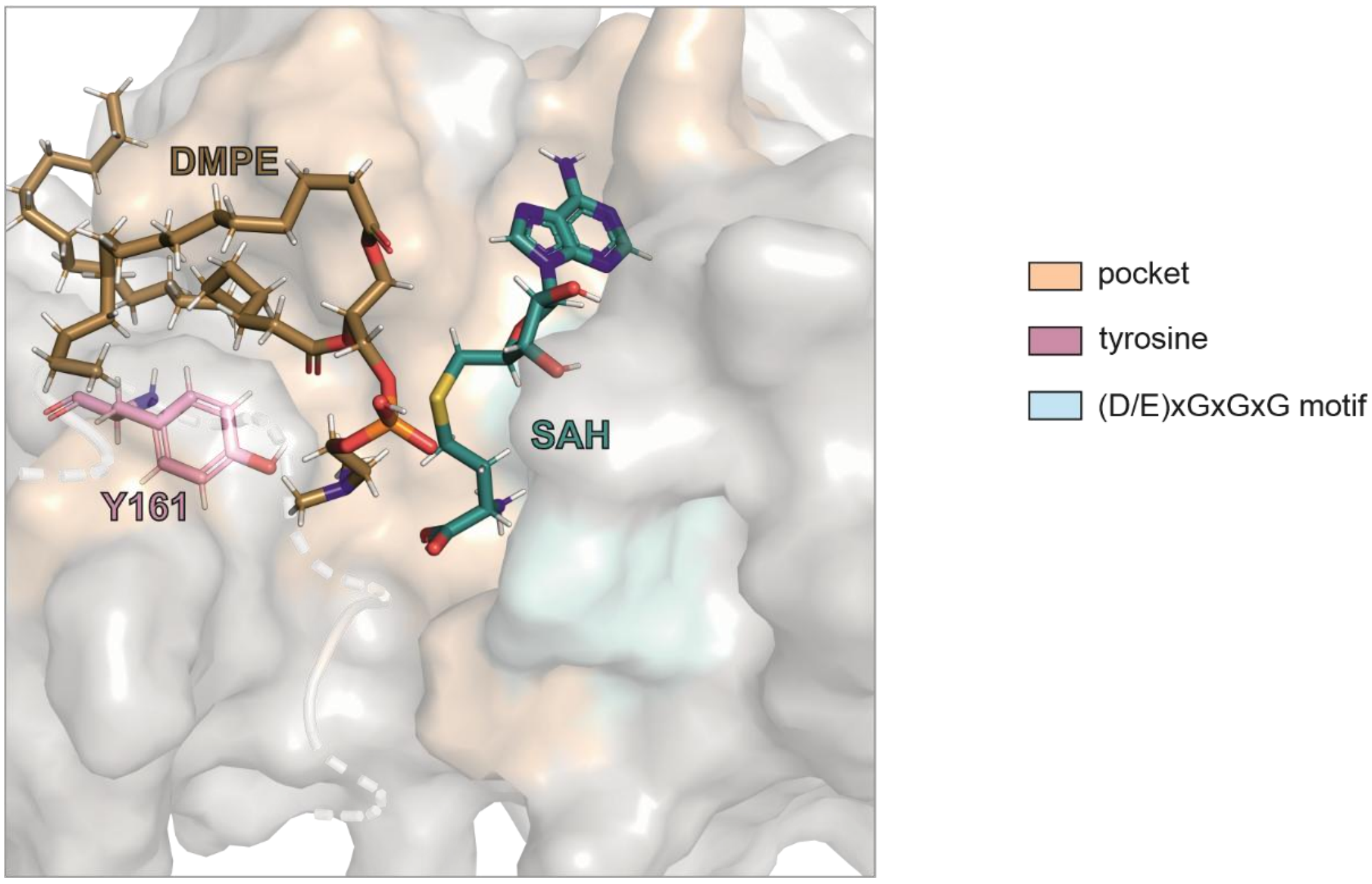

Figure S10. Substrate docking of DMPE based on the crystal structure of *At*PmtA co-crystallized with SAH. YASARA molecular modeling program was used to perform the docking [16,46]. For substrate docking, a simulation cell of 5x5x5 Å around putative lipid-binding site was defined. Tyrosine 161 was listed as one of the interacting residues in the docking with DMPE.

Table S1. Membrane phospholipid composition of *E. coli* NiCo21 (DE3) expressing *Rt*PmtA variants. Relative phospholipid levels were quantified by band intensity using ImageJ software [38] with the total lipid amount of each sample normalized to 100%. Samples showing an empty vector (EV)-like lipid profile were assigned 0% for DMPE and PC. Remaining percentages up to 100% represent other lipid species.

***Rt*PmtA variant**	**DMPE [%]**	**PC [%]**	**other lipids [%]**
EV	0	0	100
WT	11	22	67
Y10A	10	7	83
Y10F	12	9	79
Y10S	11	8	81
Y17A	0	0	100
Y17F	11	8	81
Y17S	0	0	100
Y10F/Y17F	0	0	100
Y63A	12	18	70
Y63F	10	24	66
Y63S	11	15	74
H117A	4	0	96
H117F	13	16	71
H147A	8	2	90
H147F	8	5	87
D18N	8	19	73
D73N	0	0	100
D99N	12	19	69
E160Q	14	13	73
W13A	13	11	76
F21A	0	0	100

Table S2. Quantification of methylated PE-products from *in vitro* activity assays of *Rt*PmtA variants using PE, MMPE and DMPE as substrates in the presence of PG. Relative phospholipid amounts were determined by band intensity measurements using the ImageJ software [38] with the total lipid amount of each sample normalized to 100%. After determination of DMPE and PC amounts, the remaining percentages up to 100% represent other lipid species.

**Condition**	***Rt*PmtA variant**	**DMPE [%]**	**PC [%]**	**other lipids [%]**
PE/PG	WT	8	5	87
	Y10A	6	0	94
	Y10F	0	0	100
	Y10S	0	0	100
	Y17A	0	0	100
	Y17F	0	0	100
	Y17S	0	0	100
	H117A	0	0	100
	H117F	5	2	93
	H147A	0	0	100
	H147F	0	0	100
	D73N	0	0	100
	E160Q	9	2	89
	W13A	5	2	93
	F21A	0	0	100
MMPE/PG	WT	14	5	81
	Y10A	13	4	83
	Y10F	8	0	92
	Y10S	7	0	93
	Y17A	0	0	100
	Y17F	8	1	91
	Y17S	0	0	100
	H117A	8	3	89
	H117F	13	3	84
	H147A	7	0	93
	H147F	4	2	94
	D73N	0	0	100
	E160Q	13	2	85
	W13A	12	3	85
	F21A	5	3	92
DMPE/PG	WT	-	8	92
	Y10A	-	6	94
	Y10F	-	4	96
	Y10S	-	2	98
	Y17A	-	4	96
	Y17F	-	3	97
	Y17S	-	0	100

	H117A	-	3	97
	H117F	-	5	95
	H147A	-	2	98
	H147F	-	3	97
	D73N	-	0	100
	E160Q	-	7	93
	W13A	-	5	95
	F21A	-	9	91

Table S3. Secondary structure analysis of *Rt*PmtA variants using BeStSel™ online tool [41–43]. H: alpha-helices, A: antiparallel beta-sheets, T: turns and O: other elements of secondary structure.

**[%]**	**WT**	**Y10A**	**Y10F**	**Y10S**	**Y17A**	**Y17F**	**Y17S**	**Y10F/Y17F**	**H117A**	**H117F**	**H147A**	**H147F**	**D73N**	**E160Q**	**W13A**	**F21A**
**H**	0.8	1.2	1	1.5	0.7	0.9	1.8	2.9	0.9	0.7	0.6	0.8	0	1	0.5	0.5
**A**	36	35.9	35.9	35.6	36	35.5	35.7	36.5	35.6	36	35.8	35.7	36.5	35.5	36.2	36
**T**	15.9	15.6	15.6	15.6	15.8	15.9	15.5	15.3	15.6	15.7	15.8	15.7	16.1	15.5	16	15.9
**O**	47.3	47.4	47.4	47.4	47.6	47.6	47	45.3	47.9	47.5	47.7	47.7	47.4	47.9	47.4	47.6

Table S4. Membrane phospholipid composition of *E. coli* NiCo21 (DE3) expressing *At*PmtA variants. Relative phospholipid amounts were determined by band intensity measurements using the ImageJ software [38] with the total lipid amount of each sample normalized to 100%. Samples showing an empty vector (EV)-like lipid profile were assigned 0% for DMPE and PC. Remaining percentages up to 100% represent other lipid species.

***At*PmtA variant**	**DMPE [%]**	**PC [%]**	**other lipids [%]**
EV	0	0	100
WT	6	12	82
∆N30	0	0	100
Y85A	7	14	79
Y85F	7	13	80
Y85S	7	13	80
Y161A	0	0	100
Y161F	4	7	89
Y161S	0	0	100
E84Q	0	0	100
D106N	7	12	81

Table S5. Quantification of methylated PE-products from *in vitro* activity assays of *At*PmtA variants using PE, MMPE and DMPE as substrates in the presence of PG. Relative phospholipid amounts were determined by band intensity measurements using the ImageJ software [38] with the total lipid amount of each sample normalized to 100%. After determination of DMPE and PC amounts, the remaining percentages up to 100% represent other lipid species.

**Condition**	***At*PmtA variant**	**DMPE [%]**	**PC [%]**	**other lipids [%]**
PE/PG	WT	6	4	90
	ΔN30	0	0	100
	Y161A	0	0	100
	Y161F	0	0	100
	Y161S	0	0	100
	E84Q	0	0	100
MMPE/PG	WT	9	8	83
	ΔN30	0	0	100
	Y161A	0	0	100
	Y161F	0	0	100
	Y161S	0	0	100
	E84Q	0	0	100
DMPE/PG	WT	-	13	87
	ΔN30	-	0	100
	Y161A	-	3	97
	Y161F	-	6	94
	Y161S	-	4	96
	E84Q	-	0	100

Table S6. Secondary structure analysis of *At*PmtA variants using BeStSel™ online tool [41–43]. H: alpha-helices, A: antiparallel beta-sheets, T: turns and O: other elements of secondary structure.

	**WT**	**ΔN30**	**Y161A**	**Y161F**	**Y161S**	**E84Q**
**[%]**						
**H**	4.4	2.5	2.6	4.3	2.9	3.8
**A**	34.5	35.5	34.9	34.6	34.5	34.4
**T**	14.5	15.1	15.1	14.4	15	14.6
**O**	46.6	46.9	47.5	46.7	47.6	47.1

Table S7. Strains and plasmid constructs used in this study.

**Strain**	**Description**	**Source or reference**
Escherichia coli NEB 5-alpha	Cloning host	New England Biolabs
Escherichia coli NiCo21 (DE3)	Host for gene overexpression	New England Biolabs
**Plasmid construct**		
pET28b (EV)	Km^R, high-copy number, N- and C-terminal 6xHis-tag expression vector	Novagen, Darmstadt,Germany
***Rt*PmtA WT** (pBO7022)	*R. thermophilum Rt*PmtA (ruthe_01610) in pET28b, N-terminal His-tag	[51]
***Rt*PmtA Y10A** (pBO8114)	*Rt*PmtA Y10A, pET28b, N-terminal His-tag	This study
***Rt*PmtA Y10F** (pBO8472)	*Rt*PmtA Y10F, pET28b, N-terminal His-tag	This study
***Rt*PmtA Y10S** (pBO8459)	*Rt*PmtA Y10S, pET28b, N-terminal His-tag	This study
***Rt*PmtA W13A** (pBO8473)	*Rt*PmtA W13A, pET28b, N-terminal His-tag	This study
***Rt*PmtA Y17A** (pBO8115)	*Rt*PmtA Y17A, pET28b, N-terminal His-tag	This study
***Rt*PmtA Y17F** (pBO8128)	*Rt*PmtA Y17F, pET28b, N-terminal His-tag	This study
***Rt*PmtA Y17S** (pBO8468)	*Rt*PmtA Y17S, pET28b, N-terminal His-tag	This study
***Rt*PmtA Y10F/Y17F** (pBO8150)	*Rt*PmtA Y10F/Y17F, pET28b, N-terminal His-tag	This study
***Rt*PmtA F21A** (pBO8455)	*Rt*PmtA F21A, pET28b, N-terminal His-tag	This study
***Rt*PmtA Y63A** (pBO8126)	*Rt*PmtA Y63A, pET28b, N-terminal His-tag	This study
***Rt*PmtA Y63F** (pBO8127)	*Rt*PmtA Y63F, pET28b, N-terminal His-tag	This study
***Rt*PmtA Y63S** (pBO8458)	*Rt*PmtA Y63S, pET28b, N-terminal His-tag	This study
***Rt*PmtA D18N** (pBO8456)	*Rt*PmtA D18N, pET28b, N-terminal His-tag	This study
***Rt*PmtA D73N** (pBO8470)	*Rt*PmtA D73N, pET28b, N-terminal His-tag	This study
***Rt*PmtA D99N** (pBO8469)	*Rt*PmtA D99N, pET28b, N-terminal His-tag	This study
***Rt*PmtA H117A** (pBO8465)	*Rt*PmtA H117A, pET28b, N-terminal His-tag	This study
***Rt*PmtA H117F** (pBO8466)	*Rt*PmtA H117F, pET28b, N-terminal His-tag	This study
***Rt*PmtA H147A** (pBO8467)	*Rt*PmtA H147A, pET28b, N-terminal His-tag	This study
***Rt*PmtA H147F** (pBO8463)	*Rt*PmtA H147F, pET28b, N-terminal His-tag	This study
***Rt*PmtA E160Q** (pBO8139)	*Rt*PmtA E160Q, pET28b, N-terminal His-tag	This study
***At*PmtA WT** (pBO832)	*A. tumefaciens At*PmtA (atu0300) in pET28b, N-terminal His-tag	[10]
***At*PmtA** ΔN30 (pBO3751)	*At*PmtA lacking first thirty amino acids at its N-terminus, pET28b, N-terminal His-tag	[11]
***At*PmtA Y85A** (pBO8135)	*At*PmtA Y85A, pET28b, N-terminal His-tag	This study
***At*PmtA Y85F** (pBO8134)	*At*PmtA Y85F, pET28b, N-terminal His-tag	This study
***At*PmtA Y85S** (pBO8133)	*At*PmtA Y85S, pET28b, N-terminal His-tag	This study
***At*PmtA E84Q** (pBO8136)	*At*PmtA E84Q, pET28b, N-terminal His-tag	This study
***At*PmtA Y161A** (pBO8105)	*At*PmtA Y161A, pET28b, N-terminal His-tag	This study
***At*PmtA Y161F** (pBO8131)	*At*PmtA Y161F, pET28b, N-terminal His-tag	This study
***At*PmtA Y161S** (pBO8132)	*At*PmtA Y161S, pET28b, N-terminal His-tag	This study

Table S8. Oligonucleotides used in this study.

Protein variants	Forward Primer (5´- 3´)	Reverse Primer (5´- 3´)
**of *Rt*PmtA**		
**Y10A**	CGAGGCAAGCGCTCGCCGCTGG	ACGGCCTCGATCTCCATAT
**Y10F**	CGAGGCAAGCTTCCGCCGCTGGG	ACGGCCTCGATCTCCATTCAG
**Y10S**	CGAGGCAAGCAGCCGCCGCTGGG	ACGGCCTCGATCTCCATTC
**Y17A**	GGCGCCGATCGCTGACCGCACC	CAGCGGCGATAGCTTGCC
**Y17F**	GCGCCGATCTTCGATCGCACCTTCG	CCAGCGGCGATAGCTTGC
**Y17S**	GGCGCCGATCTCTGACCGCACCT	CAGCGGCGATAGCTTGCC
**Y10F/Y17F**	GGCGCCGATCTTTGACCGCACCT	CAGCGGCGGAAGCTTGC
**Y63A**	GCTGCCCCATGCGGCGCCCCATATCCG	GCGAGCCCCGTGCC
**Y63F**	GCTGCCCCATTTTGCGCCCCATATCCG	GCGAGCCCCGTGCC
**Y63S**	GCTGCCCCATAGCGCGCCCCATATC	GCGAGCCCCGTGCCC
**D18N**	GCCGATCTATAACCGCACCTTCG	GCCCAGCGGCGATAG
**D73N**	GACCGGCATCAACGCCAGCGAGG	ACGCGGATATGGGGC
**D99N**	GCGGATCATGAATGCCCGCTCGC	AGGGATTCGACCGTGGGC
**H117A**	GGCGGCCATGGCGGTCCTTTCCG	ACCACATCGAAGCTTTC
**H117F**	GGCGGCCATGTTTGTCCTTTCCG	ACCACATCGAAGCTTTC
**H147A**	CACGGTCACGGCGTTCGCCGTCGAGGG	ACAGCCTGTCCGCCG
**H147F**	CACGGTCACGTTTTTCGCCGTCGAG	ACAGCCTGTCCGCCG
**E160Q**	GGCCGCGCTGCAGCGGGCGATGG	CGCGCGCCCCGCCCC
**W13A**	CTATCGCCGCGCGGCGCCGATCTATG	CTTGCCTCGACGGCCTCG
**F21A**	TGACCGCACCGCGGGTGCCGTCA	TAGATCGGCGCCCAG
**of *At*PmtA**		
**Y85A**	GGCCATCGAAGCGTCGACTGATTTCTACAATC	GTCAGGCTCTCCGGC
**Y85F**	GGCCATCGAATTTTCGACTGATTTC	GTCAGGCTCTCCGGC
**Y85S**	GGCCATCGAAAGCTCGACTGATTTCTACAATC	GTCAGGCTCTCCGGC
**E84Q**	GACGGCCATCCAATATTCGACTG	AGGCTCTCCGGCTTG
**Y161A**	GTACAGATCTCCGCCGGTCCGATTTC	TACGGGTCGGCCGTGCG
**Y161F**	ACAGATATCCTTTGGTCCGATTTCCCCG	ACTACGGGTCGGCCG
**Y161S**	ACAGATATCCAGCGGTCCGATTTCCCC	ACTACGGGTCGGCCG
